# ﻿The ornithological collection of the Zoological Museum of Babeș-Bolyai University, Cluj-Napoca, Romania – Part 1: the catalogue of bird skin specimens

**DOI:** 10.3897/zookeys.1102.79102

**Published:** 2022-05-19

**Authors:** Gergely Osváth, Edgár Papp, Zoltán Benkő, Zsolt Kovács

**Affiliations:** 1 Museum of Zoology, Babeș-Bolyai University, Cluj-Napoca, Romania; 2 Evolutionary Ecology Group, Hungarian Department of Biology and Ecology, Babeş-Bolyai University, Cluj-Napoca, Romania; 3 Milvus Group Bird and Nature Protection Association, Târgu Mureș, Romania; 4 Romanian Ornithological Society/BirdLife Romania, Cluj-Napoca, Romania

**Keywords:** Aves, biodiversity, bird skin, museum, ornithology, ornithological collections

## Abstract

This paper reviews the bird skin collection housed in the Zoological Museum of Babeș-Bolyai University, Cluj-Napoca, Romania. The collection includes 925 specimens, belonging to 193 species from 53 families and 20 orders, collected between 1859 and 2021. Due to its historical background and the presence of rare species, it is considered to be one of most important ornithological collections in Eastern Europe. Such a collection can serve as a basis for valuable ornithological studies. Furthermore, a map representation with new distribution data for bird species is provided, which represents a source of information for the status of the avifauna of the Carpathian basin in the 19^th^ and 20^th^ centuries.

## ﻿Introduction

Museum collections are important primary data sources for addressing fundamental questions in morphology, systematics, biogeography and biodiversity conservation (Causey et al. 2004; Vágási et al. 2016; Bartoccioni 2017; Pap et al. 2017, 2019, 2020; Osváth et al. 2018, 2020; Meineke et al. 2019). Collections generally comprise specimens from different time periods and areas; thus, well-labelled preserved specimens provide information on how the environment and species distribution has changed over extended time periods (Solow and Roberts 2006; MacLean et al. 2019; Gotelli et al. 2021). The importance of keeping specimens in collections and making them publicly available is increasing, particularly in the case of old collections, which cover long time periods (Roselaar 2003; Waeber et al. 2017; Mikula et al. 2018).

**Figure 1.**
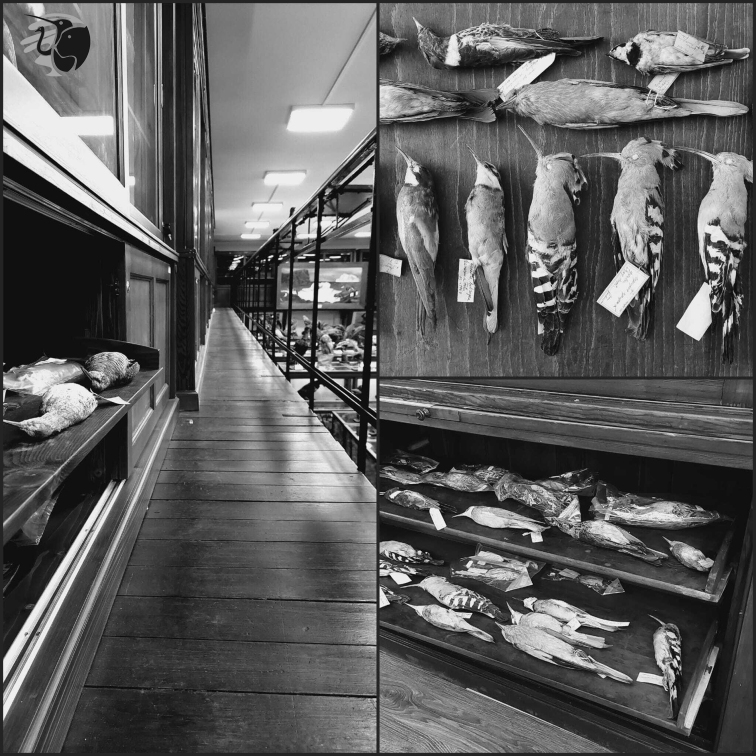
Bird skin specimens from the ornithological collection of the Zoological Museum of Babeș-Bolyai University, Cluj-Napoca, Romania.

An important ornithological collection is held in the Zoological Museum of Babeș-Bolyai University, Cluj-Napoca, Romania (Fig. 1). The collection is unique in the region in many ways: it covers a long time span, it contains a variety of species, belonging to different families and orders, and it is composed of the work of several naturalists and employees of the museum. Bird skins account for approximately half of the total ornithological collection and they were only partially catalogued. Information about the collection had been published, particularly in the early stages of the museum’s history (e.g., Herman 1865, 1868, 1869; Apáthy 1910b, 1910a, 1911; Filipașcu et al. 1965; Filipașcu 1966), but the revision of the full collection had not been carried out and all specimen data had not been made public until now.

Hence, our aim was to systematically verify the species identification of the bird skin specimens in the Zoological Museum of Babeș-Bolyai University ornithological collection to provide a catalogue of these birds, including the following information: list of species, number of specimens per species, up to date taxonomic nomenclature, and collection data (date, location, collector).

## ﻿Materials and methods

We examined each bird skin in the collection and the data cards documenting the identification, locality, date, sex and catalogue number. After this, we checked the species identification of specimens, and we determined the sex and age of birds, where possible. The scientific name and the taxonomy of birds was updated following Handbook of the Birds of the World and BirdLife International Taxonomic Checklist v. 5 (2020).

Some specimens lacked a data card but had old inventory numbers. Therefore, in order to maximise the information content of these specimens, we researched contemporary museum registers and early museum-related reports for data. Following identification, all available specimen data were entered into the updated digital catalogue of the bird skin collection. All collection locality information was georeferenced.

### ﻿Collection summary

**Figure 2.**
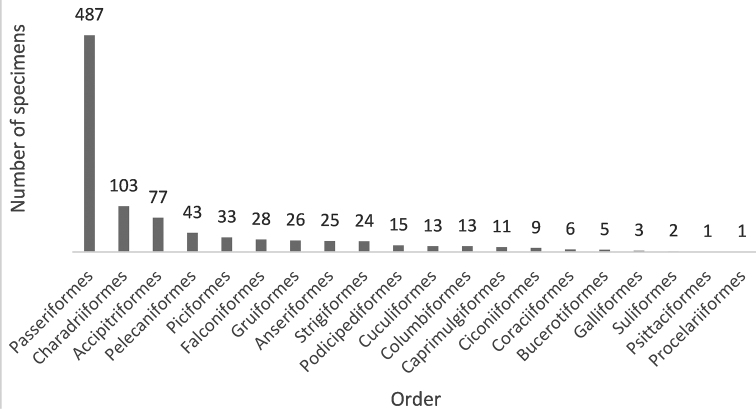
Total number of bird skin specimens per order represented in the ornithological collection of the Zoological Museum of Babeș-Bolyai University, Cluj-Napoca, Romania.

In total, we identified 925 specimens in the scientific bird skin collection housed in the Zoological Museum of Babeș-Bolyai University (Table 1; for detailed catalogue see Suppl. material 1: Table S1), belonging to 193 species from 53 families and 20 orders (Fig. 2). The orders with the most specimens were Passeriformes (487), Charadriiformes (103), Accipitriformes (77), Pelecaniformes (43), Piciformes (33), Falconiformes (28), and Gruiformes (26). Twelve orders (Anseriformes, Strigiformes, Podicipediformes, Columbiformes, Cuculiformes, Caprimulgiformes, Bucerotiformes, Ciconiiformes, Coraciiformes, Galliformes, Suliformes, Procelariiformes, and Psittaciformes) were represented by 25 or fewer specimens (Fig. 2).

**Table 1.** Bird skin specimens held by the Zoological Museum of Babeș-Bolyai University, Romania, including their updated identification and scientific name, locality of collection, name of collector (surname, given name), date of collection, and sex and age of birds. The full catalogue of bird skin collection is provided in the Suppl. material 1: Table S1. A blank cell indicates no available data.

Species	Location	Name of collector	Data	Sex	Age	Order
*Acanthisflammea*	Cluj-Napoca (CJ)	Zwörner Sándor	23.01.1904	M	adult	Passeriformes
*Acanthisflammea*	Cluj-Napoca (CJ)	Korodi-Gál János	16.02.1973	M	juvenile	Passeriformes
*Acanthisflammea*	Cluj-Napoca (CJ)	Korodi-Gál János	16.02.1973	M	adult	Passeriformes
*Acanthisflammea*	Cluj-Napoca (CJ)	Korodi-Gál János	16.02.1973	M	juvenile	Passeriformes
*Acanthisflammea*	Cluj-Napoca (CJ)	Korodi-Gál János	16.02.1973	F	adult	Passeriformes
*Acanthisflammea*	Cluj-Napoca (CJ)	Korodi-Gál János	16.02.1973	F	adult	Passeriformes
*Acanthisflammea*	Cluj-Napoca (CJ)	Korodi-Gál János	16.02.1973	F	adult	Passeriformes
*Acanthisflammea*	Cluj-Napoca (CJ)	Korodi-Gál János	16.02.1973	F	adult	Passeriformes
*Acanthisflammea*	Cluj-Napoca (CJ)	Korodi-Gál János	16.02.1973	M	adult	Passeriformes
*Accipitergentilis*	Aghireș (CJ)	Führer Lajos	29.02.1906	M	adult	Accipitriformes
*Accipitergentilis*	Micești (CJ)	Führer Lajos	03.10.1908	F	juvenile	Accipitriformes
*Accipitergentilis*	Cluj-Napoca (CJ)	Führer Lajos	02.07.1905	M	juvenile	Accipitriformes
*Accipitergentilis*	Turea (CJ)	Führer Lajos	26.08.1905	M	juvenile	Accipitriformes
*Accipitergentilis*				M	adult	Accipitriformes
*Accipitergentilis*				F	juvenile	Accipitriformes
*Accipitergentilis*				M	juvenile	Accipitriformes
*Accipiternisus*	Cluj-Napoca (CJ)	Ajtai K. Gyula	xx.08.1892	F	juvenile	Accipitriformes
*Accipiternisus*	Turea (CJ)	Führer Lajos	26.08.1905	F	juvenile	Accipitriformes
*Accipiternisus*	Turea (CJ)	Führer Lajos	15.07.1905	F	adult	Accipitriformes
*Accipiternisus*		Zwörner Sándor	03.10.1903	M	juvenile	Accipitriformes
*Acrocephalusarundinaceus*	Apahida (CJ)	Führer Lajos	xx.05.1911	F	adult	Passeriformes
*Acrocephalusarundinaceus*	Apahida (CJ)	Führer Lajos	xx.05.1911	M	adult	Passeriformes
*Acrocephalusarundinaceus*	Apahida (CJ)	Führer Lajos	xx.05.1911	F	adult	Passeriformes
*Acrocephalusarundinaceus*	Apahida (CJ)	Führer Lajos	xx.05.1911	M	adult	Passeriformes
*Acrocephalusarundinaceus*	Apahida (CJ)	Führer Lajos	xx.05.1911	M	adult	Passeriformes
*Acrocephalusarundinaceus*	Apahida (CJ)	Führer Lajos	xx.09.1909	F	adult	Passeriformes
*Acrocephalusarundinaceus*	Apahida (CJ)	Führer Lajos	xx.09.1909	M	adult	Passeriformes
*Acrocephaluspalustris*	Cluj-Napoca (CJ)	Führer Lajos	xx.05.1911		adult	Passeriformes
*Acrocephaluspalustris*	Florești (CJ)	Führer Lajos	xx.05.1913	M	adult	Passeriformes
*Acrocephaluspalustris*						Passeriformes
*Acrocephaluspalustris*						Passeriformes
*Acrocephaluspalustris*	Cluj-Napoca (CJ)	Führer Lajos	xx.05.1911			Passeriformes
*Acrocephaluspalustris*						Passeriformes
*Acrocephalusschoenabaenus*	Apahida (CJ)	Führer Lajos	xx.10.1909	M		Passeriformes
*Acrocephalusschoenabaenus*	Apahida (CJ)	Führer Lajos	xx.10.1909	M	juvenile	Passeriformes
*Acrocephalusschoenabaenus*	Apahida (CJ)	Führer Lajos	xx.10.1909	F		Passeriformes
*Acrocephalusschoenabaenus*			xx.xx.1911		adult	Passeriformes
*Acrocephalusschoenabaenus*					adult	Passeriformes
*Acrocephalusschoenabaenus*			xx.xx.1911			Passeriformes
*Acrocephalusschoenabaenus*						Passeriformes
*Acrocephalusscirpaceus*						Passeriformes
*Actitishypoleucos*	Cluj-Napoca (CJ)	Führer Lajos	xx.06.1911	M	juvenile	Charadriiformes
*Actitishypoleucos*	Hortobágy (HU)		xx.04.1907	F		Charadriiformes
*Actitishypoleucos*	Geaca (CJ)	Herman Ottó	xx.04.1867	F	adult	Charadriiformes
*Actitishypoleucos*	Gilău (CJ)	Führer Lajos	xx.05.1911	F	adult	Charadriiformes
*Actitishypoleucos*	Gilău (CJ)	Führer Lajos	xx.05.1911	F	adult	Charadriiformes
*Actitishypoleucos*	Gilău (CJ)	Führer Lajos	xx.06.1911	F	juvenile	Charadriiformes
*Actitishypoleucos*	Gilău (CJ)	Führer Lajos	xx.06.1911	M	adult	Charadriiformes
*Actitishypoleucos*	Mociu (CJ)	Führer Lajos	xx.10.1909	M	juvenile	Charadriiformes
*Actitishypoleucos*	Mociu (CJ)	Führer Lajos	xx.10.1909	M	adult	Charadriiformes
*Actitishypoleucos*	Mociu (CJ)	Führer Lajos	xx.10.1909	F	adult	Charadriiformes
*Actitishypoleucos*	Mociu (CJ)	Führer Lajos	xx.06.1910	M		Charadriiformes
*Actitishypoleucos*	Răscruci (CJ)	Führer Lajos	xx.11.1909	M	juvenile	Charadriiformes
*Actitishypoleucos*	Răscruci (CJ)	Führer Lajos	xx.11.1909	F	juvenile	Charadriiformes
*Actitishypoleucos*	Szentgothárd (HU)	Ajtai K. Gyula	23.05.1910	M	adult	Charadriiformes
*Actitishypoleucos*					adult	Charadriiformes
*Aegithaloscaudatus*	Cluj-Napoca (CJ)	Führer Lajos	xx.01.1910	F	adult	Passeriformes
*Aegithaloscaudatus*	Cluj-Napoca (CJ)	Ajtai K. Gyula	20.02.1910	M	adult	Passeriformes
*Aegithaloscaudatus*	Cluj-Napoca (CJ)	Führer Lajos	xx.12.1909	M	adult	Passeriformes
*Aegithaloscaudatus*	Cluj-Napoca (CJ)	Führer Lajos	21.12.1902	F	adult	Passeriformes
*Aegithaloscaudatus*	Feleacu (CJ)	Führer Lajos	xx.05.1911	M	adult	Passeriformes
*Aegithaloscaudatus*	Micești (CJ)	Führer Lajos	xx.11.1909	F	adult	Passeriformes
*Aegithaloscaudatus*	Micești (CJ)	Führer Lajos	xx.01.1909	M	adult	Passeriformes
*Aegithaloscaudatus*					adult	Passeriformes
*Aegithaloscaudatus*					adult	Passeriformes
*Aegithaloscaudatus*					adult	Passeriformes
*Aegypiusmonachus*	Bucium (SJ)		24.06.1903	F	adult	Accipitriformes
*Alaudaarvensis*	Apahida (CJ)	Führer Lajos	xx.10.1909	M		Passeriformes
*Alaudaarvensis*	Apahida (CJ)	Führer Lajos	xx.10.1909	M		Passeriformes
*Alaudaarvensis*	Cluj-Napoca (CJ)	Vincze Ferencz	14.03.1965	M	adult	Passeriformes
*Alaudaarvensis*						Passeriformes
*Alaudaarvensis*						Passeriformes
*Alcedoatthis*	Florești (CJ)	Führer Lajos	16.08.1912	M		Coraciiformes
*Anasacuta*	Ocna Mureș (AB)	Führer Lajos	17.03.1903	M	adult	Anseriformes
*Anascrecca*	Geaca (CJ)	Vincze Ferencz	30.09.1971	F		Anseriformes
*Anascrecca*	Geaca (CJ)	Vincze Ferencz	08.10.1971	M	juvenile	Anseriformes
*Anascrecca*	Geaca (CJ)	Vincze Ferencz	08.10.1971	F		Anseriformes
*Anasplatyrhynchos*	Hăghig (CV)	Führer Lajos	11.01.1903	F		Anseriformes
*Anasplatyrhynchos*				F		Anseriformes
*Anseralbifrons*	Cefa (BH)	Vincze Ferencz	19.12.1970	F	juvenile	Anseriformes
*Anseralbifrons*					juvenile	Anseriformes
*Anserfabalis*	Hortobágy (HU)	Teleky O.	xx.04.1907	F		Anseriformes
*Anserfabalis*						Anseriformes
*Anthuscampestris*	Suatu (CJ)	Führer Lajos	xx.06.1911	M		Passeriformes
*Anthuscervinus*		Führer Lajos				Passeriformes
*Anthusspinoletta*	Răscruci (CJ)	Führer Lajos	xx.10.1909	M		Passeriformes
*Anthustrivialis*	Apahida (CJ)	Führer Lajos	xx.10.1909	F		Passeriformes
*Anthustrivialis*	Apahida (CJ)	Führer Lajos	xx.10.1909	F		Passeriformes
*Anthustrivialis*	Cuzăplac (SJ)	Kómis Lajos	18.10.1913	M		Passeriformes
*Anthustrivialis*	Florești (CJ)	Führer Lajos	xx.03.1913			Passeriformes
*Anthustrivialis*						Passeriformes
*Aquilaheliaca*	Sibiu (SB)	Führer Lajos	xx.08.1907	F	adult	Accipitriformes
*Aquilaheliaca*	Sibiu (SB)	Führer Lajos	xx.08.1907	M	juvenile	Accipitriformes
*Ardeaalba*	Mociu (CJ)	Führer Lajos	xx.10.1909	M		Pelecaniformes
*Ardeaalba*	Mociu (CJ)	Führer Lajos	xx.10.1909	F		Pelecaniformes
*Ardeacinerea*	Băgara (CJ)	Führer Lajos	xx.04.1910	F	adult	Pelecaniformes
*Ardeacinerea*	Cefa (BH)	Vincze Ferencz	23.06.1970	M	adult	Pelecaniformes
*Ardeacinerea*	Cefa (BH)	Vincze Ferencz	23.06.1970	M	adult	Pelecaniformes
*Ardeacinerea*	Cefa (BH)	Vincze Ferencz	23.06.1970	M	adult	Pelecaniformes
*Ardeacinerea*	Cefa (BH)	Vincze Ferencz	23.06.1970	F	juvenile	Pelecaniformes
*Ardeacinerea*	Cefa (BH)	Vincze Ferencz	23.06.1970	F	juvenile	Pelecaniformes
*Ardeacinerea*	Mociu (CJ)	Führer Lajos	xx.04.1910	M	adult	Pelecaniformes
*Ardeacinerea*	Răscruci (CJ)	Führer Lajos	xx.10.1909	F	juvenile	Pelecaniformes
*Ardeacinerea*	Țaga (CJ)	Ajtai K. Gyula	03.05.1910	F	immatur	Pelecaniformes
*Ardeacinerea*			xx.xx.1911		adult	Pelecaniformes
*Ardeacinerea*			xx.xx.1911		adult	Pelecaniformes
*Ardeacinerea*			xx.xx.1911		adult	Pelecaniformes
*Ardeacinerea*			xx.xx.1911		adult	Pelecaniformes
*Ardeapurpurea*	Băgara (CJ)	Führer Lajos	xx.04.1910	F	adult	Pelecaniformes
*Ardeapurpurea*	Geaca (CJ)	Vincze Ferencz	15.09.1972	F	juvenile	Pelecaniformes
*Ardeapurpurea*	Geaca (CJ)	Führer Lajos	xx.xx.1911		adult	Pelecaniformes
*Ardeapurpurea*					juvenile	Pelecaniformes
*Ardeolaralloides*	Cefa (BH)	Vincze Ferencz	24.06.1970	M	adult	Pelecaniformes
*Asioflammeus*	Apahida (CJ)	Führer Lajos	xx.03.1911	F	adult	Strigiformes
*Asioflammeus*	Borș (BH)	Führer Lajos	15.01.1906	F	adult	Strigiformes
*Asioflammeus*	Borș (BH)	Führer Lajos	15.01.1906	M	adult	Strigiformes
*Asioflammeus*	Cluj-Napoca (CJ)	Kómis Lajos	30.11.1913	F	adult	Strigiformes
*Asioflammeus*					adult	Strigiformes
*Asioflammeus*				M	adult	Strigiformes
*Asioflammeus*				F	adult	Strigiformes
*Asioflammeus*				F	adult	Strigiformes
*Asioflammeus*					adult	Strigiformes
*Asioflammeus*					adult	Strigiformes
*Asioflammeus*				F	adult	Strigiformes
*Asiootus*	Baia Mare (MM)	Sitar Cristian	xx.12.2010		adult	Strigiformes
*Asiootus*	Cefa (BH)	Vincze Ferencz	19.12.1970	F	adult	Strigiformes
*Asiootus*	Hortobágy (HU)	Nagy Jenő	xx.04.1907	M	adult	Strigiformes
*Asiootus*	Przewtoka (UA)	Kómis Lajos	06.04.1916	M	adult	Strigiformes
*Asiootus*	Turda (CJ)		xx.04.1893		adult	Strigiformes
*Asiootus*					adult	Strigiformes
*Athenenoctua*		Führer Lajos	xx.xx.1911		adult	Strigiformes
*Athenenoctua*	Sărmășel - Gară (MS)	Osváth Gergely	14.05.2020		adult	Strigiformes
*Aythyaferina*	Cefa (BH)	Vincze Ferencz	24.06.1970	F	juvenile	Anseriformes
*Aythyaferina*	Geaca (CJ)	Vincze Ferencz	04.06.1970	M	adult	Anseriformes
*Aythyanyroca*	Apahida (CJ)	Führer Lajos	xx.09.1909	F		Anseriformes
*Aythyanyroca*	Cefa (BH)	Vincze Ferencz	24.06.1970	F		Anseriformes
*Aythyanyroca*	Geaca (CJ)	Vincze Ferencz	03.06.1970	F		Anseriformes
*Aythyanyroca*	Geaca (CJ)	Vincze Ferencz	30.09.1971	F		Anseriformes
*Aythyanyroca*	Geaca (CJ)	Vincze Ferencz	03.06.1970	F	adult	Anseriformes
*Aythyanyroca*					adult	Anseriformes
*Bombycillagarrulus*	Cluj-Napoca (CJ)	Zwörner Sándor	18.12.1903	M		Passeriformes
*Bombycillagarrulus*	Cluj-Napoca (CJ)	Führer Lajos	10.11.1903	M		Passeriformes
*Bombycillagarrulus*	Cluj-Napoca (CJ)	Führer Lajos	10.11.1903	M		Passeriformes
*Bombycillagarrulus*	Cluj-Napoca (CJ)	Führer Lajos	10.11.1903	F		Passeriformes
*Bombycillagarrulus*				M		Passeriformes
*Bombycillagarrulus*				M		Passeriformes
*Bombycillagarrulus*				M		Passeriformes
*Bombycillagarrulus*				M		Passeriformes
*Bombycillagarrulus*				M		Passeriformes
*Bombycillagarrulus*				M		Passeriformes
*Buteobuteo*	Apahida (CJ)	Führer Lajos	xx.07.1912	F		Accipitriformes
*Buteobuteo*	Apahida (CJ)	Führer Lajos	xx.07.1912	M		Accipitriformes
*Buteobuteo*	Ardeal		xx.12.1863	M		Accipitriformes
*Buteobuteo*	Borșa (CJ)	Führer Lajos	29.03.1906	M		Accipitriformes
*Buteobuteo*	Cluj-Napoca (CJ)	Fülöp Herman	25.02.1960	M		Accipitriformes
*Buteobuteo*	Cluj-Napoca (CJ)		xx.02.1913	M		Accipitriformes
*Buteobuteo*	Cluj-Napoca (CJ)	Führer Lajos	19.07.1905	M		Accipitriformes
*Buteobuteo*	Cluj-Napoca (CJ)	Ajtai K. Gyula	11.10.1909	F		Accipitriformes
*Buteobuteo*	Cristian (BV)	Lánczy I.	25.01.1903	F		Accipitriformes
*Buteobuteo*	Dej (CJ)	Zwörner Sándor	08.01.1904			Accipitriformes
*Buteobuteo*	Făgăraș (BV)	Zwörner Sándor	01.05.1903	M		Accipitriformes
*Buteobuteo*	Florești (CJ)		xx.xx.1913	F		Accipitriformes
*Buteobuteo*	Florești (CJ)	Führer Lajos	xx.10.1913	M		Accipitriformes
*Buteobuteo*	Grădina Zoologică din Târgu Mureș	Bereczki Boldizsár	15.02.1985			Accipitriformes
*Buteobuteo*	Grădina Zoologică din Târgu Mureș	Bereczki Boldizsár	15.02.1985			Accipitriformes
*Buteobuteo*	Miskolc (HU)	Herman Ottó?	xx.12.1863	F		Accipitriformes
*Buteobuteo*						Accipitriformes
*Buteobuteo*						Accipitriformes
*Buteobuteo*						Accipitriformes
*Buteobuteo*						Accipitriformes
*Buteobuteo*						Accipitriformes
*Buteobuteo*						Accipitriformes
*Buteobuteo*						Accipitriformes
*Buteobuteo*						Accipitriformes
*Buteobuteo*		Fülöp Herman				Accipitriformes
*Buteobuteo*						Accipitriformes
*Buteobuteo*	Delnița (CJ)	Miklós Réka, Osváth Gergely	12.10.2020		immatur	Accipitriformes
*Buteolagopus*	Apahida (CJ)		xx.02.1913	F	adult	Accipitriformes
*Buteolagopus*	Cluj-Napoca (CJ)	Führer Lajos	07.02.1905	F	adult	Accipitriformes
*Buteolagopus*	Cluj-Napoca (CJ)	Herman Ottó?	xx.xx.1865	M	adult	Accipitriformes
*Buteolagopus*				M	adult	Accipitriformes
*Buteolagopus*				F?	adult	Accipitriformes
*Buteolagopus*				F	adult	Accipitriformes
*Buteorufinus*	Europa de Sud-Est			M		Accipitriformes
*Buteorufinus*	Răscruci (CJ)	Führer Lajos	xx.10.1909	M		Accipitriformes
*Calidrisminuta*	Apahida (CJ)	Führer Lajos	xx.05.1911	M	adult	Charadriiformes
*Calidrisminuta*					adult	Charadriiformes
*Calidrisminuta*	Someșeni (CJ)	Führer Lajos	xx.05.1911	M	adult	Charadriiformes
*Calidrispugnax*	Cătina (CJ)	Zwörner Sándor	11.05.1904	F	adult	Charadriiformes
*Calidrispugnax*	Hortobágy (HU)	Nagy Jenő	xx.04.1907	F	adult	Charadriiformes
*Calidrispugnax*				M	adult	Charadriiformes
*Caprimulguseuropaeus*	Cluj-Napoca (CJ)	Führer Lajos	xx.04.1911	M		Caprimulgiformes
*Caprimulguseuropaeus*	Cluj-Napoca (CJ)	Führer Lajos	10.10.1912	F		Caprimulgiformes
*Caprimulguseuropaeus*	Cluj-Napoca (CJ)	Führer Lajos	xx.04.1911	M		Caprimulgiformes
*Caprimulguseuropaeus*	Cluj-Napoca (CJ)	Führer Lajos	xx.04.1911	M		Caprimulgiformes
*Caprimulguseuropaeus*	Cluj-Napoca (CJ)	Führer Lajos	xx.04.1911	M		Caprimulgiformes
*Caprimulguseuropaeus*	Feleacu (CJ)	Führer Lajos	xx.04.1911	F		Caprimulgiformes
*Caprimulguseuropaeus*	Florești (CJ)	Vincze Ferencz	18.05.1970	F		Caprimulgiformes
*Caprimulguseuropaeus*						Caprimulgiformes
*Caprimulguseuropaeus*						Caprimulgiformes
*Caprimulguseuropaeus*						Caprimulgiformes
*Caprimulguseuropaeus*						Caprimulgiformes
*Cardueliscarduelis*	Pădureni (CJ)	Vincze Ferencz	02.02.1985		adult	Passeriformes
*Certhiafamiliaris*	Cuzăplac (SJ)	Kómis Lajos	xx.04.1912	F		Passeriformes
*Certhiafamiliaris*	Feleacu (CJ)	Führer Lajos	xx.05.1911	M		Passeriformes
*Certhiafamiliaris*	Feleacu (CJ)	Führer Lajos	xx.04.1911	M	adult	Passeriformes
*Certhiafamiliaris*						Passeriformes
*Certhiafamiliaris*						Passeriformes
*Certhiafamiliaris*						Passeriformes
*Charadriusalexandrinus*	Hortobágy (HU)	Teleki I.	xx.04.1907	F		Charadriiformes
*Charadriusdubius*	Cluj-Napoca (CJ)	Zwörner Sándor	15.04.1904	F		Charadriiformes
*Charadriusdubius*	Florești (CJ)	Führer Lajos	xx.04.1913	M		Charadriiformes
*Charadriusdubius*	Cluj-Napoca (CJ)	Führer Lajos	xx.05.1911	F		Charadriiformes
*Charadriusdubius*	Cluj-Napoca (CJ)	Führer Lajos	xx.05.1911	M		Charadriiformes
*Charadriusdubius*	Cluj-Napoca (CJ)	Zwörner Sándor	18.04.1904	M		Charadriiformes
*Chlidoniashybrida*	Apahida (CJ)	Führer Lajos	xx.09.1909	F	adult	Charadriiformes
*Chlidoniashybrida*	Apahida (CJ)	Führer Lajos	xx.09.1909	F	adult	Charadriiformes
*Chlidoniashybrida*	Apahida (CJ)	Führer Lajos	xx.09.1909	M	adult	Charadriiformes
*Chlidoniashybrida*	Apahida (CJ)	Führer Lajos	xx.10.1909	M	adult	Charadriiformes
*Chlidoniashybrida*	Apahida (CJ)	Führer Lajos	xx.10.1909	F	adult	Charadriiformes
*Chlidoniasniger*					juvenile	Charadriiformes
*Chlidoniasniger*	Apahida (CJ)	Führer Lajos	xx.09.1909	M	adult	Charadriiformes
*Chlidoniasniger*	Brașov (BV)	Zwörner Sándor	08.07.1903	M	adult	Charadriiformes
*Chlidoniasniger*	Geaca (CJ)	Vincze Ferencz	30.08.1971	F	juvenile	Charadriiformes
*Chlorischloris*	Cluj-Napoca (CJ)		04.03.1985	M	adult	Passeriformes
*Chlorischloris*	Cuzăplac (SJ)	Kómis Lajos	16.10.1913	F	adult	Passeriformes
*Chlorischloris*				F	adult	Passeriformes
*Chlorischloris*				F	adult	Passeriformes
*Ciconiaciconia*	Dezmir (CJ)	Fülöp Herman	18.08.1958	F	juvenile	Ciconiiformes
*Ciconiaciconia*	Grădina Zoologică din Turda	Vincze Ferencz	10.11.1971	M	adult	Ciconiiformes
*Ciconiaciconia*	Someșeni (CJ)	Vincze Ferencz	10.10.1972	M	adult	Ciconiiformes
*Ciconiaciconia*			xx.xx.1911		adult	Ciconiiformes
*Ciconiaciconia*			xx.xx.1911		adult	Ciconiiformes
*Ciconiaciconia*					adult	Ciconiiformes
*Ciconianigra*	Răscruci (CJ)	Führer Lajos	xx.10.1909	F	juvenile	Ciconiiformes
*Ciconianigra*				F	juvenile	Ciconiiformes
*Ciconianigra*		Führer Lajos	xx.xx.1911	M	adult	Ciconiiformes
*Cincluscinclus*	Bradu (NT)	Vincze Ferencz	10.05.1971	F		Passeriformes
*Cincluscinclus*	Cluj-Napoca (CJ)	Ajtai K. Gyula	18.11.1909	M		Passeriformes
*Cincluscinclus*	Someșul rece (CJ)	Vincze Ferencz	06.09.1970	M	juvenile	Passeriformes
*Cincluscinclus*			xx.xx.1912			Passeriformes
*Cincluscinclus*			xx.xx.1912			Passeriformes
*Cincluscinclus*			xx.xx.1912			Passeriformes
*Cincluscinclus*						Passeriformes
*Cincluscinclus*	Mara (MM)	Deák József	10.08.1961			Passeriformes
*Circaetusgallicus*	Sibiu (SB)	Führer Lajos	xx.07.1907	M		Accipitriformes
*Circusaeruginosus*	Florești (CJ)	Führer Lajos	xx.xx.1911	M	juvenile	Accipitriformes
*Circusaeruginosus*	Hăghig (CV)	Führer Lajos	01.05.1903	F	adult	Accipitriformes
*Circuscyaneus*				F	adult	Accipitriformes
*Clangaclanga*	Bonțida (CJ)	Führer Lajos	xx.02.1910	F	juvenile	Accipitriformes
*Clangaclanga*	Bonțida (CJ)	Führer Lajos	xx.02.1910	M?	juvenile	Accipitriformes
*Clangaclanga*	Gilău (CJ)	Führer Lajos	23.03.1896	F		Accipitriformes
*Clangapomarina*	Apahida (CJ)	Führer Lajos	xx.04.1911	F		Accipitriformes
*Clangapomarina*	Cuzăplac (SJ)	Führer Lajos	20.01.1914	F	juvenile	Accipitriformes
*Clangapomarina*	Feleacu (CJ)	Führer Lajos	xx.05.1911	F		Accipitriformes
*Clangapomarina*					adult	Accipitriformes
*Clangapomarina*					adult	Accipitriformes
*Clangapomarina*					adult	Accipitriformes
*Coccothraustescoccothraustes*	Someșeni (CJ)		12.09.1972	M		Passeriformes
*Columbaoenas*	Cluj-Napoca (CJ)	Führer Lajos	xx.06.1911	F	adult	Columbiformes
*Columbaoenas*	Cordoș (MS)	Führer Lajos	xx.07.1911	M	adult	Columbiformes
*Columbaoenas*	Feleacu (CJ)	Führer Lajos	xx.04.1911	M	adult	Columbiformes
*Columbaoenas*					adult	Columbiformes
*Columbaoenas*					adult	Columbiformes
*Columbaoenas*		Führer Lajos	xx.xx.1911		adult	Columbiformes
*Coraciasgarrulus*	Bicaz (NT)	Führer Lajos	xx.07.1910	F		Coraciiformes
*Corvuscornix*	Someșeni (CJ)	Neuwirth János	24.03.1903	M		Passeriformes
*Corvusfrugilegus*						Passeriformes
*Corvusfrugilegus*						Passeriformes
*Corvusmonedula*	Cluj-Napoca (CJ)	Zwörner Sándor	02.02.1904	M	adult	Passeriformes
*Corvusmonedula*	Cluj-Napoca (CJ)	Zwörner Sándor	19.11.1903	F		Passeriformes
*Corvusmonedula*	Turea (CJ)	Führer Lajos	23.08.1903	M	adult	Passeriformes
*Corvusmonedula*				M		Passeriformes
*Coturnixcoturnix*	Apahida (CJ)	Führer Lajos	xx.09.1909	M	juvenile	Galliformes
*Coturnixcoturnix*	Gilău (CJ)	Führer Lajos	xx.09.1909	M		Galliformes
*Crexcrex*	Apahida (CJ)	Zwörner Sándor	05.06.1903	F		Gruiformes
*Crexcrex*	Cluj-Napoca (CJ)		05.09.1902	M		Gruiformes
*Crexcrex*	Geaca (CJ)	Herman Ottó	xx.04.1867	M	adult	Gruiformes
*Cuculuscanorus*	Aghireș (CJ)	Führer Lajos	xx.09.1909	M	adult	Cuculiformes
*Cuculuscanorus*	Aghireș (CJ)	Führer Lajos	xx.09.1909	M	adult	Cuculiformes
*Cuculuscanorus*	Ceahlău (NT)	Vincze Ferencz	08.05.1971	M	adult	Cuculiformes
*Cuculuscanorus*	Cluj-Napoca (CJ)	Vincze Ferencz	25.09.1971	F	juvenile	Cuculiformes
*Cuculuscanorus*	Cluj-Napoca (CJ)	Führer Lajos	xx.10.1912	M	adult	Cuculiformes
*Cuculuscanorus*	Cluj-Napoca (CJ)	Führer Lajos	xx.04.1911	M	adult	Cuculiformes
*Cuculuscanorus*	Cluj-Napoca (CJ)	Führer Lajos	xx.04.1911	M	adult	Cuculiformes
*Cuculuscanorus*	Cluj-Napoca (CJ)	Führer Lajos	xx.04.1911	M	adult	Cuculiformes
*Cuculuscanorus*	Cluj-Napoca (CJ)	Führer Lajos	xx.xx.1912	M	juvenile	Cuculiformes
*Cuculuscanorus*	Feleacu (CJ)	Führer Lajos	xx.04.1911	F	adult	Cuculiformes
*Cuculuscanorus*	Feleacu (CJ)	Führer Lajos	xx.04.1911	M	adult	Cuculiformes
*Cuculuscanorus*	Pănade (AB)	Führer Lajos	xx.09.1909	M	adult	Cuculiformes
*Cuculuscanorus*	Turnu Roșu (SB)	Ajtai K. Gyula	12.04.1910	M	adult	Cuculiformes
*Cyanistescaeruleus*	Cluj-Napoca (CJ)	Führer Lajos	18.11.1913	M		Passeriformes
*Cyanistescaeruleus*	Cluj-Napoca (CJ)	Führer Lajos	xx.09.1912	M		Passeriformes
*Cyanistescaeruleus*	Cluj-Napoca (CJ)	Führer Lajos	xx.03.1910	M		Passeriformes
*Cyanistescaeruleus*	Cluj-Napoca (CJ)	Ajtai K. Gyula	11.02.1910	M		Passeriformes
*Cyanistescaeruleus*	Florești (CJ)	Neuwirth János	26.03.1903	M		Passeriformes
*Cyanistescaeruleus*	Micești (CJ)	Führer Lajos	xx.11.1909	M		Passeriformes
*Cyanistescaeruleus*						Passeriformes
*Cyanistescaeruleus*						Passeriformes
*Cyanistescaeruleus*						Passeriformes
*Delichonurbicum*	Apahida (CJ)	Führer Lajos	xx.09.1909	M		Passeriformes
*Delichonurbicum*	Apahida (CJ)	Führer Lajos	xx.09.1909	M		Passeriformes
*Delichonurbicum*	Apahida (CJ)	Führer Lajos	xx.09.1909	M		Passeriformes
*Dendrocoposleucotos*	Almașu (SJ)	Kómis Lajos	15.01.1914	M	adult	Piciformes
*Dendrocoposmajor*	Cluj-Napoca (CJ)	Zwörner Sándor	14.02.1903	F	adult	Piciformes
*Dendrocoposmajor*	Plesca (SJ)	Führer Lajos	26.03.1903	F	adult	Piciformes
*Dendrocoposmajor*				M	adult	Piciformes
*Dendrocoposmajor*				F	adult	Piciformes
*Dendrocoposmajor*	Alba Iulia (AB)	Savu George, Osváth Gergely	23.02.2018	M	adult	Piciformes
*Dryobatesminor*	Feleacu (CJ)	Führer Lajos	xx.04.1909	M		Piciformes
*Dryobatesminor*	Feleacu (CJ)	Führer Lajos	xx.04.1909	M		Piciformes
*Dryocopusmartius*	Colibița (BN)	Vincze Ferencz	06.05.1971	M		Piciformes
*Dryocopusmartius*	Măguri-Răcătău (CJ)	Vincze Ferencz	03.08.1970	F		Piciformes
*Dryocopusmartius*						Piciformes
*Egrettagarzetta*	Cefa (BH)	Vincze Ferencz	26.07.1970	M		Pelecaniformes
*Egrettagarzetta*	Cefa (BH)	Vincze Ferencz	23.0.06.1970	m		Pelecaniformes
*Emberizacitrinella*	Cluj-Napoca (CJ)	Fülöp Herman	31.01.1960	M		Passeriformes
*Emberizacitrinella*	Someșeni (CJ)	Vincze Ferencz	25.11.1972	M		Passeriformes
*Emberizacitrinella*						Passeriformes
*Emberizacitrinella*					immatur	Passeriformes
*Emberizacitrinella*				M?	immatur	Passeriformes
*Eremophilaalpestris*	Florești (CJ)	Führer Lajos	xx.04.1911	M	adult	Passeriformes
*Eremophilaalpestris*	Florești (CJ)	Führer Lajos	xx.04.1911	M	adult	Passeriformes
*Erithacusrubecula*	Cluj-Napoca (CJ)	Führer Lajos	xx.08.1905	M		Passeriformes
*Erithacusrubecula*	Cluj-Napoca (CJ)	Führer Lajos	xx.10.1909	M		Passeriformes
*Erithacusrubecula*	Cluj-Napoca (CJ)	Führer Lajos	xx.10.1909	M		Passeriformes
*Erithacusrubecula*	Cluj-Napoca (CJ)	Führer Lajos	xx.10.1909	M		Passeriformes
*Erithacusrubecula*	Cluj-Napoca (CJ)	Führer Lajos	xx.09.1912	M		Passeriformes
*Erithacusrubecula*	Cluj-Napoca (CJ)	Führer Lajos	xx.09.1912	M		Passeriformes
*Erithacusrubecula*	Făget (CJ)	Führer Lajos	xx.10.1909	F		Passeriformes
*Erithacusrubecula*	Florești (CJ)	Führer Lajos	xx.03.1913	M		Passeriformes
*Erithacusrubecula*			xx.10.1910	F		Passeriformes
*Erithacusrubecula*						Passeriformes
*Estrildatroglodytes*	Apahida (CJ)	Führer Lajos	xx.09.1912	M	adult	Passeriformes
*Eupsaltriaaustralis*	Victoria (AU)	Gasilemaine	xx.07.1897			Passeriformes
*Falcocherrug*	Bonțida (CJ)	Führer Lajos	xx.04.1911	F		Falconiformes
*Falcocherrug*	Bonțida (CJ)	Führer Lajos	xx.04.1911	F	adult	Falconiformes
*Falcocherrug*	Răscruci (CJ)	Führer Lajos	xx.02.1910	M		Falconiformes
*Falcocherrug*	Răscruci (CJ)	Führer Lajos	xx.11.1909	M		Falconiformes
*Falcocherrug*				M	adult	Falconiformes
*Falcocherrug*				F	adult	Falconiformes
*Falcocherrug*				M	adult	Falconiformes
*Falcocherrug*				F	juvenile	Falconiformes
*Falcocherrug*				F	adult	Falconiformes
*Falcocherrug*				M	adult	Falconiformes
*Falcocherrug*				F	adult	Falconiformes
*Falcocherrug*	Răscruci (CJ)		xx.03.1911	M	adult	Falconiformes
*Falconaumanni*	Cluj-Napoca (CJ)	Führer Lajos	01.09.1905	M	adult	Falconiformes
*Falcoperegrinus*	Aghireș (CJ)		xx.03.1910	M	adult	Falconiformes
*Falcoperegrinus*				F	juvenile	Falconiformes
*Falcosubbuteo*	Cluj-Napoca (CJ)	Herman Ottó?	xx.xx.1864	M	juvenile	Falconiformes
*Falcosubbuteo*	Cluj-Napoca (CJ)	Herman Ottó?	xx.xx.1864	F	adult	Falconiformes
*Falcosubbuteo*	Cluj-Napoca (CJ)	Herman Ottó?	xx.xx.1864	M	adult	Falconiformes
*Falcosubbuteo*	Geaca (CJ)	Vincze Ferencz	07.09.1970	M	adult	Falconiformes
*Falcosubbuteo*	Turea (CJ)		27.08.1965	M	adult	Falconiformes
*Falcosubbuteo*					adult	Falconiformes
*Falcosubbuteo*					adult	Falconiformes
*Falcotinnunculus*	Cluj-Napoca (CJ)	Zwörner Sándor	26.04.1903	M	adult	Falconiformes
*Falcotinnunculus*	Florești (CJ)	Vincze Ferencz	13.08.1970	M	adult	Falconiformes
*Falcotinnunculus*	Galiția de Est		29.04.1916	F		Falconiformes
*Falcotinnunculus*				F	juvenile	Falconiformes
*Falcotinnunculus*				M	juvenile	Falconiformes
*Falcovespertinus*	Răscruci (CJ)	Führer Lajos	xx.05.1910	F	adult	Falconiformes
*Ficedulaalbicollis*	Feleacu (CJ)	Führer Lajos	xx.05.1911	M	adult	Passeriformes
*Ficedulaalbicollis*	Gilău (CJ)	Führer Lajos	xx.05.1911	M	adult	Passeriformes
*Ficedulaalbicollis*	Gilău (CJ)	Führer Lajos	xx.05.1911	M	adult	Passeriformes
*Ficedulaalbicollis*	Gilău (CJ)	Führer Lajos	xx.06.1911	M	adult	Passeriformes
*Ficedulaalbicollis*				M	adult	Passeriformes
*Ficedulahypoleuca*	Aghireș (CJ)	Führer Lajos	xx.09.1909	M	adult	Passeriformes
*Ficedulahypoleuca*	Cluj-Napoca (CJ)	Führer Lajos	xx.09.1909	M	adult	Passeriformes
*Ficedulahypoleuca*	Cluj-Napoca (CJ)	Führer Lajos	xx.08.1912	M	adult	Passeriformes
*Ficedulahypoleuca*	Făget (CJ)	Führer Lajos	xx.09.1909	M	juvenile	Passeriformes
*Ficedulahypoleuca*	Feleacu (CJ)	Führer Lajos	xx.05.1911	M	adult	Passeriformes
*Ficedulahypoleuca*	Gilău (CJ)	Führer Lajos	xx.05.1911	F	adult	Passeriformes
*Ficedulahypoleuca*	Gilău (CJ)	Führer Lajos	xx.05.1911	M	adult	Passeriformes
*Ficedulahypoleuca*	Gilău (CJ)	Führer Lajos	xx.06.1911	M	adult	Passeriformes
*Ficedulahypoleuca*				M	adult	Passeriformes
*Ficedulaparva*	Făget (CJ)	Führer Lajos	xx.09.1909	F	juvenile	Passeriformes
*Ficedulaparva*	Făget (CJ)	Führer Lajos	xx.09.1909	M	juvenile	Passeriformes
*Ficedulaparva*	Făget (CJ)	Führer Lajos	xx.05.1911		juvenile	Passeriformes
*Fringillamontifringilla*				F		Passeriformes
*Fringillamontifringilla*				F		Passeriformes
*Fringillamontifringilla*				M		Passeriformes
*Fringillamontifringilla*				M		Passeriformes
*Fulicaatra*	Geaca (CJ)	Herman Ottó	xx.03.1867			Gruiformes
*Fulicaatra*	Geaca (CJ)	Herman Ottó	xx.03.1867	M		Gruiformes
*Fulicaatra*	Geaca (CJ)	Vincze Ferencz	15.10.1971	M		Gruiformes
*Fulicaatra*	Geaca (CJ)	Vincze Ferencz	15.10.1971	F		Gruiformes
*Fulicaatra*		Vincze Ferencz	22.11.1973			Gruiformes
*Fulicaatra*		Vincze Ferencz	22.11.1973			Gruiformes
*Fulmarusglacialis*					adult	Procelariiformes
*Galeridacristata*	Apahida (CJ)	Führer Lajos	27.10.1912	M		Passeriformes
*Galeridacristata*	Cluj-Napoca (CJ)	Zwörner Sándor	25.04.1904	M		Passeriformes
*Galeridacristata*	Cluj-Napoca (CJ)	Führer Lajos	20.02.1913	M		Passeriformes
*Galeridacristata*	Cluj-Napoca (CJ)	Führer Lajos	13.03.1903	M		Passeriformes
*Galeridacristata*	Țaga (CJ)	Fülöp Herman	31.01.1960	F		Passeriformes
*Galeridacristata*	Țaga (CJ)	Fülöp Herman	31.01.1960	M		Passeriformes
*Galeridacristata*						Passeriformes
*Gallinulachloropus*	Apahida (CJ)	Führer Lajos	xx.11.1909	M	adult	Gruiformes
*Gallinulachloropus*	Sucutard (CJ)	Vincze Ferencz	20.10.1971		juvenile	Gruiformes
*Gallinulachloropus*	Țaga (CJ)	Ajtai K. Gyula	02.05.1910	M	adult	Gruiformes
*Gallinulachloropus*					adult	Gruiformes
*Gallinagogallinago*	Aghireș (CJ)	Führer Lajos	xx.09.1909	M		Charadriiformes
*Gallinagogallinago*	Apahida (CJ)	Führer Lajos	xx.08.1912		juvenile	Charadriiformes
*Gallinagogallinago*	Mociu (CJ)	Führer Lajos	xx.11.1909	F		Charadriiformes
*Gallinagogallinago*	Mociu (CJ)	Führer Lajos	xx.11.1909	M		Charadriiformes
*Gallinagogallinago*						Charadriiformes
*Gypaetusbarbatus*	Tibet (Asia)			M	adult	Accipitriformes
*Gypsfulvus*		Führer Lajos	xx.07.1907	F	juvenile	Accipitriformes
*Haliaeetusalbicilla*	Răscruci (CJ)	Führer Lajos	xx.11.1909	F	juvenile	Accipitriformes
*Haliaeetusalbicilla*	Zau de Câmpie (MS)	Führer Lajos	xx.08.1907	F	juvenile	Accipitriformes
*Haliaeetusalbicilla*	Zau de Câmpie (MS)	Führer Lajos	xx.08.1907	F	immatur	Accipitriformes
*Haliaeetusalbicilla*	Zau de Câmpie (MS)	Führer Lajos	xx.07.1907	F	subadult	Accipitriformes
*Haliaeetusalbicilla*	Zau de Câmpie (MS)	Führer Lajos	xx.08.1907	F	immatur	Accipitriformes
*Hieraaetuspennatus*	Răscruci (CJ)	Führer Lajos	xx.10.1909	M	juvenile	Accipitriformes
*Hieraaetuspennatus*	Răscruci (CJ)	Führer Lajos	xx.10.1909	M	juvenile	Accipitriformes
*Hieraaetuspennatus*	Răscruci (CJ)	Führer Lajos	xx.10.1909	M	juvenile	Accipitriformes
*Hieraaetuspennatus*	Răscruci (CJ)	Führer Lajos	xx.10.1909	M		Accipitriformes
*Hieraaetuspennatus*						Accipitriformes
*Himantopushimantopus*	Mociu (CJ)	Führer Lajos	xx.11.1909	F	juvenile	Charadriiformes
*Himantopushimantopus*						Charadriiformes
*Hippolaisicterina*	Cluj-Napoca (CJ)	Korodi-Gál János	28.05.1965	M	adult	Passeriformes
*Hippolaisicterina*	Cluj-Napoca (CJ)	Korodi-Gál János	28.05.1965	F	adult	Passeriformes
*Hippolaisicterina*	Cluj-Napoca (CJ)	Korodi-Gál János	28.05.1965	M	adult	Passeriformes
*Hippolaisicterina*	Cluj-Napoca (CJ)	Korodi-Gál János	28.05.1965	M	adult	Passeriformes
*Hirundorustica*	Apahida (CJ)	Führer Lajos	xx.09.1909	F	adult	Passeriformes
*Hirundorustica*	Apahida (CJ)	Führer Lajos	xx.09.1909	F	adult	Passeriformes
*Hirundorustica*	Apahida (CJ)	Führer Lajos	xx.09.1909	F	juvenile	Passeriformes
*Hirundorustica*	Apahida (CJ)	Führer Lajos	xx.09.1909	F	adult	Passeriformes
*Hirundorustica*	Cluj-Napoca (CJ)	Führer Lajos	xx.09.1912	1y		Passeriformes
*Hirundorustica*	Florești (CJ)	Führer Lajos	xx.05.1913	F	adult	Passeriformes
*Hirundorustica*	Florești (CJ)	Führer Lajos	xx.05.1913	F	adult	Passeriformes
*Hirundorustica*				F		Passeriformes
*Idunapallida*	Cluj-Napoca (CJ)	Korodi-Gál János	28.05.1965	F	adult	Passeriformes
*Ixobrychusminutus*	Răscruci (CJ)	Führer Lajos	xx.10.1909	M	adult	Pelecaniformes
*Jynxtorquilla*	Florești (CJ)	Führer Lajos	xx.04.1913	M		Piciformes
*Jynxtorquilla*	Gilău (CJ)	Führer Lajos	xx.09.1909	F		Piciformes
*Jynxtorquilla*	Viștea (CJ)	Führer Lajos	xx.09.1909	F		Piciformes
*Laniuscollurio*	Baciu (CJ)	Ajtai K. Gyula	26.05.1910	M		Passeriformes
*Laniuscollurio*	Cluj-Napoca (CJ)	Führer Lajos	01.08.1905	M		Passeriformes
*Laniuscollurio*	Cluj-Napoca (CJ)	Führer Lajos	27.07.1905	F		Passeriformes
*Laniuscollurio*	Cluj-Napoca (CJ)		xx.07.1891	M		Passeriformes
*Laniuscollurio*	Cluj-Napoca (CJ)	Führer Lajos	xx.05.1910	M		Passeriformes
*Laniuscollurio*	Cluj-Napoca (CJ)	Vincze Ferencz	03.08.1970	F		Passeriformes
*Laniuscollurio*	Cluj-Napoca (CJ)	Führer Lajos	xx.08.1912	F	juvenile	Passeriformes
*Laniuscollurio*	Cluj-Napoca (CJ)	Führer Lajos	xx.10.1909		juvenile	Passeriformes
*Laniuscollurio*	Cuzăplac (SJ)		16.07.1913	F		Passeriformes
*Laniuscollurio*	Gilău (CJ)	Führer Lajos	xx.10.1909		juvenile	Passeriformes
*Laniuscollurio*	Gilău (CJ)	Führer Lajos	xx.10.1909		juvenile	Passeriformes
*Laniuscollurio*				M		Passeriformes
*Laniuscollurio*				M		Passeriformes
*Laniusexcubitor*	Cuzăplac (SJ)	Kómis Lajos	16.12.1913	M	adult	Passeriformes
*Laniusexcubitor*	Florești (CJ)	Führer Lajos	xx.12.1912		juvenile	Passeriformes
*Laniusexcubitor*	Florești (CJ)	Führer Lajos	xx.12.1912	M	adult	Passeriformes
*Laniusexcubitor*	Someșeni (CJ)	Vincze Ferencz	22.10.1972	M	adult	Passeriformes
*Laniusminor*	Baciu (CJ)	Führer Lajos	xx.05.1910	M	adult	Passeriformes
*Laniusminor*						Passeriformes
*Laruscanus*	Apahida (CJ)	Führer Lajos	xx.02.1910	M	immatur	Charadriiformes
*Laruscanus*	Hortobágy (HU)	Nagy Jenő	xx.04.1907	F	adult	Charadriiformes
*Laruscanus*	Zau de Câmpie (MS)		xx.03.1911	F	adult	Charadriiformes
*Laruscanus*	Zau de Câmpie (MS)		xx.03.1911	M	adult	Charadriiformes
*Larusfuscus*	Someșeni (CJ)		xx.10.1902	F	juvenile	Charadriiformes
*Larusmarinus*					adult	Charadriiformes
*Larusmichahellis*	Apahida (CJ)	Führer Lajos	xx.11.1909	F		Charadriiformes
*Larusmichahellis*	Apahida (CJ)	Führer Lajos	xx.11.1909	F		Charadriiformes
*Larusmichahellis*					immatur	Charadriiformes
*Larusmichahellis*					adult	Charadriiformes
*Larusridibundus*	Apahida (CJ)	Führer Lajos	xx.10.1909	F	adult	Charadriiformes
*Larusridibundus*	Apahida (CJ)	Führer Lajos	xx.10.1909	F	juvenile	Charadriiformes
*Larusridibundus*	Apahida (CJ)	Führer Lajos	xx.11.1909	M	juvenile	Charadriiformes
*Larusridibundus*	Dezmir (CJ)	Führer Lajos	xx.11.1909	F	juvenile	Charadriiformes
*Larusridibundus*	Mociu (CJ)	Führer Lajos	xx.11.1909	M	juvenile	Charadriiformes
*Larusridibundus*	Mociu (CJ)	Führer Lajos	xx.06.1910	F	adult	Charadriiformes
*Larusridibundus*	Mociu (CJ)	Führer Lajos	xx.06.1910	M	adult	Charadriiformes
*Larusridibundus*	Mociu (CJ)	Führer Lajos	xx.06.1910	M	adult	Charadriiformes
*Larusridibundus*					adult	Charadriiformes
*Larusridibundus*	Zau de Câmpie (MS)		xx.04.1911		adult	Charadriiformes
*Larusridibundus*	Zau de Câmpie (MS)		xx.04.1911		immatur	Charadriiformes
*Larusridibundus*					juvenile	Charadriiformes
*Larusridibundus*					adult	Charadriiformes
*Leiopicusmedius*	Aghireș (CJ)	Führer Lajos	xx.09.1909	M		Piciformes
*Leiopicusmedius*	Feleacu (CJ)	Führer Lajos	xx.05.1911	M		Piciformes
*Leiopicusmedius*	Feleacu (CJ)	Führer Lajos	xx.05.1911	F		Piciformes
*Leiopicusmedius*	Feleacu (CJ)	Führer Lajos	xx.05.1911	M		Piciformes
*Leiopicusmedius*	Feleacu (CJ)	Führer Lajos	xx.05.1911	M		Piciformes
*Leiopicusmedius*	Feleacu (CJ)	Führer Lajos	xx.05.1911	M		Piciformes
*Leiopicusmedius*	Feleacu (CJ)	Führer Lajos	xx.05.1911	M		Piciformes
*Leiopicusmedius*	Micești (CJ)	Führer Lajos	xx.11.1909	F		Piciformes
*Leiopicusmedius*	Micești (CJ)	Führer Lajos	xx.11.1909	M		Piciformes
*Leiopicusmedius*	Micești (CJ)	Führer Lajos	xx.11.1909	M		Piciformes
*Leiopicusmedius*	Unguraș (CJ)	Führer Lajos	xx.09.1909	F		Piciformes
*Leiopicusmedius*		Führer Lajos	xx.09.1909	F		Piciformes
*Lichenostomusfuscus*	Victoria (AU)	Gasilemaine	xx.07.1897			Passeriformes
*Limosalimosa*	Someșeni (CJ)	Vincze Ferencz	26.07.1973	M	juvenile	Charadriiformes
*Limosalimosa*	Ungaria (HU)		xx.04.1911	M	adult	Charadriiformes
*Linariacannabina*	Cuzăplac (SJ)	Kómis Lajos	xx.07.1913	F		Passeriformes
*Linariacannabina*	Cuzăplac (SJ)	Kómis Lajos	23.10.1913	M		Passeriformes
*Linariacannabina*				M		Passeriformes
*Linariacannabina*				M		Passeriformes
*Locustellaluscionides*	Apahida (CJ)	Führer Lajos	xx.05.1911			Passeriformes
*Locustellaluscionides*	Apahida (CJ)	Führer Lajos	xx.05.1911	M		Passeriformes
*Locustellaluscionides*	Apahida (CJ)	Führer Lajos	xx.05.1911	M		Passeriformes
*Locustellaluscionides*	Apahida (CJ)	Führer Lajos	xx.05.1911	M		Passeriformes
*Locustellaluscionides*	Apahida (CJ)	Führer Lajos	xx.05.1911	M		Passeriformes
*Locustellaluscionides*	Apahida (CJ)	Führer Lajos	xx.05.1911			Passeriformes
*Locustellaluscionides*						Passeriformes
*Locustellaluscionides*						Passeriformes
*Locustellaluscionides*						Passeriformes
*Locustellaluscionides*			xx.xx.1911			Passeriformes
*Locustellaluscionides*			xx.xx.1911			Passeriformes
*Loxiacurvirostra*	Cluj-Napoca (CJ)	Vincze Ferencz	13.10.1970	M	adult	Passeriformes
*Loxiacurvirostra*	Cluj-Napoca (CJ)	Vincze Ferencz	13.10.1970	F	adult	Passeriformes
*Loxiacurvirostra*	Cluj-Napoca (CJ)	Fülöp Herman	11.12.1959	F	adult	Passeriformes
*Lullulaarborea*	Feleacu (CJ)	Führer Lajos	xx.05.1911	M	adult	Passeriformes
*Lullulaarborea*	Feleacu (CJ)	Führer Lajos	xx.05.1911	F	adult	Passeriformes
*Lullulaarborea*	Feleacu (CJ)	Führer Lajos	xx.05.1911	M	adult	Passeriformes
*Lullulaarborea*	Plesca (SJ)	Neuwirth János	15.03.1903	F	adult	Passeriformes
*Lullulaarborea*					adult	Passeriformes
*Lullulaarborea*					adult	Passeriformes
*Luscinialuscinia*	Aghireș (CJ)	Führer Lajos	xx.09.1909	F		Passeriformes
*Luscinialuscinia*	Aghireș (CJ)	Führer Lajos	xx.05.1911	M	adult	Passeriformes
*Luscinialuscinia*	Cluj-Napoca (CJ)	Führer Lajos	xx.05.1911	F		Passeriformes
*Luscinialuscinia*	Cluj-Napoca (CJ)	Führer Lajos	xx.05.1911	F		Passeriformes
*Luscinialuscinia*	Cluj-Napoca (CJ)	Führer Lajos	xx.10.1909	M		Passeriformes
*Luscinialuscinia*	Cluj-Napoca (CJ)	Führer Lajos	xx.10.1909	M		Passeriformes
*Luscinialuscinia*	Făget (CJ)	Führer Lajos	xx.10.1909	M		Passeriformes
*Luscinialuscinia*	Făget (CJ)	Führer Lajos	xx.10.1909	M		Passeriformes
*Luscinialuscinia*	Făget (CJ)	Führer Lajos	xx.09.1909	F		Passeriformes
*Luscinialuscinia*	Făget (CJ)	Führer Lajos	xx.10.1909	F		Passeriformes
*Luscinialuscinia*	Răscruci (CJ)	Führer Lajos	xx.10.1909	F		Passeriformes
*Lusciniamegarhynchos*	Aghireș (CJ)	Führer Lajos	xx.05.1911	F		Passeriformes
*Lusciniamegarhynchos*	Cluj-Napoca (CJ)	Führer Lajos	xx.05.1911	M		Passeriformes
*Lusciniamegarhynchos*	Cluj-Napoca (CJ)	Führer Lajos	xx.05.1911	M		Passeriformes
*Lusciniamegarhynchos*	Cluj-Napoca (CJ)	Führer Lajos	xx.08.1912	M		Passeriformes
*Lymnocryptesminimus*	Aghireș (CJ)	Führer Lajos	xx.09.1909	F		Charadriiformes
*Lymnocryptesminimus*	Aghireș (CJ)	Führer Lajos	xx.09.1909	M		Charadriiformes
*Lymnocryptesminimus*	Răscruci (CJ)	Führer Lajos	xx.11.1909	M		Charadriiformes
*Lymnocryptesminimus*						Charadriiformes
*Lymnocryptesminimus*	Răscruci (CJ)	Führer Lajos	xx.11.1909	F		Charadriiformes
*Lymnocryptesminimus*	Răscruci (CJ)	Führer Lajos	xx.11.1909	F		Charadriiformes
*Lymnocryptesminimus*	Răscruci (CJ)	Führer Lajos	xx.11.1909	M		Charadriiformes
*Marecastrepera*	Apahida (CJ)	Führer Lajos	xx.04.1911	F		Anseriformes
*Marecastrepera*	Apahida (CJ)	Führer Lajos	xx.11.1909	M		Anseriformes
*Melopsittacusundulatus*				M	adult	Psittaciformes
*Mergusmerganser*	Cluj-Napoca (CJ)	Zwörner Sándor	15.10.1903	F	adult	Anseriformes
*Meropsapiaster*	București (B)	Vincze Ferencz	12.05.1969	M	adult	Coraciiformes
*Meropsapiaster*	Canaraua Fetei (CT)	Vincze Ferencz	12.05.1969			Coraciiformes
*Meropsapiaster*		Führer Lajos	xx.05.1911	M		Coraciiformes
*Meropsapiaster*		Führer Lajos	xx.05.1911	M		Coraciiformes
*Milvusmigrans*	Apahida (CJ)		xx.11.1911	M	juvenile	Accipitriformes
*Milvusmigrans*					juvenile	Accipitriformes
*Monticolasaxatilis*						Passeriformes
*Morusbassanus*	Norvegia	Lehne W.	xx.xx.1969		adult	Suliformes
*Morusbassanus*					juvenile	Suliformes
*Motacillaalba*	Apahida (CJ)	Führer Lajos	xx.10.1909	M		Passeriformes
*Motacillaalba*	Apahida (CJ)	Führer Lajos	xx.10.1909	F		Passeriformes
*Motacillaalba*	Apahida (CJ)	Führer Lajos	xx.09.1909	M		Passeriformes
*Motacillaalba*	Apahida (CJ)	Führer Lajos	xx.11.1909	F		Passeriformes
*Motacillaalba*	Cluj-Napoca (CJ)	Zwörner Sándor	02.04.1904	F		Passeriformes
*Motacillaalba*	Cluj-Napoca (CJ)	Führer Lajos	28.09.1912	M	juvenile	Passeriformes
*Motacillaalba*	Cluj-Napoca (CJ)	Führer Lajos	xx.09.1912	M	juvenile	Passeriformes
*Motacillaalba*	Cluj-Napoca (CJ)	Führer Lajos	xx.09.1912	M	juvenile	Passeriformes
*Motacillaalba*	Cluj-Napoca (CJ)	Führer Lajos	xx.09.1912	M	juvenile	Passeriformes
*Motacillaalba*	Cluj-Napoca (CJ)	Führer Lajos	xx.09.1912		juvenile	Passeriformes
*Motacillaalba*	Cluj-Napoca (CJ)	Führer Lajos	xx.03.1910	M		Passeriformes
*Motacillaalba*	Cluj-Napoca (CJ)	Führer Lajos	xx.01.1909	F		Passeriformes
*Motacillaalba*	Florești (CJ)	Führer Lajos	xx.04.1913	M		Passeriformes
*Motacillaalba*						Passeriformes
*Motacillaalba*				M		Passeriformes
*Motacillaalba*				M		Passeriformes
*Motacillacinerea*	Colibița (BN)	Vincze Ferencz	07.05.1971	F		Passeriformes
*Motacillacinerea*				M		Passeriformes
*Motacillacinerea*				F		Passeriformes
*Motacillacinerea*				M		Passeriformes
*Motacillacinerea*				F		Passeriformes
*Muscicapastriata*	Cluj-Napoca (CJ)	Führer Lajos	10.10.1912	M	juvenile	Passeriformes
*Muscicapastriata*	Cluj-Napoca (CJ)	Führer Lajos	xx.09.1912	F	juvenile	Passeriformes
*Muscicapastriata*	Cluj-Napoca (CJ)	Führer Lajos	xx.10.1909	M	juvenile	Passeriformes
*Muscicapastriata*	Cluj-Napoca (CJ)	Führer Lajos	xx.09.1912	M	adult	Passeriformes
*Muscicapastriata*	Cluj-Napoca (CJ)	Führer Lajos	xx.09.1912	M	juvenile	Passeriformes
*Muscicapastriata*	Cluj-Napoca (CJ)	Führer Lajos	xx.09.1912	M	juvenile	Passeriformes
*Muscicapastriata*	Cluj-Napoca (CJ)	Führer Lajos	xx.09.1912	F	juvenile	Passeriformes
*Muscicapastriata*	Cluj-Napoca (CJ)	Führer Lajos	xx.09.1912	F	juvenile	Passeriformes
*Muscicapastriata*	Cluj-Napoca (CJ)	Führer Lajos	xx.09.1912		juvenile	Passeriformes
*Muscicapastriata*	Cluj-Napoca (CJ)	Führer Lajos	xx.09.1912	F	juvenile	Passeriformes
*Myiarchuscinerascens*	California (USA)	Xantus János	xx.xx.1859	F	adult	Passeriformes
*Nucifragacaryocatactes*	Măguri-Răcătău (CJ)	Vincze Ferencz	03.08.1970	M		Passeriformes
*Nucifragacaryocatactes*						Passeriformes
*Nucifragacaryocatactes*						Passeriformes
*Nycticoraxnycticorax*	Cefa (BH)	Vincze Ferencz	19.12.1970	M	adult	Pelecaniformes
*Nycticoraxnycticorax*	Cefa (BH)	Vincze Ferencz	25.06.1970	F	juvenile	Pelecaniformes
*Nycticoraxnycticorax*	Cefa (BH)	Vincze Ferencz	25.06.1970	F	juvenile	Pelecaniformes
*Nycticoraxnycticorax*	Cefa (BH)	Vincze Ferencz	25.06.1970	M	juvenile	Pelecaniformes
*Nycticoraxnycticorax*	Cefa (BH)	Vincze Ferencz	25.06.1970	M	juvenile	Pelecaniformes
*Nycticoraxnycticorax*	Gilău (CJ)	Führer Lajos	xx.09.1909	M	adult	Pelecaniformes
*Nycticoraxnycticorax*	Mociu (CJ)	Führer Lajos	xx.05.1910	M	adult	Pelecaniformes
*Nycticoraxnycticorax*	Dej (CJ)	Führer Lajos	24.06.1903	M	adult	Pelecaniformes
*Nycticoraxnycticorax*	Dej (CJ)	Führer Lajos	17.06.1903	M	adult	Pelecaniformes
*Nycticoraxnycticorax*	Răscruci (CJ)	Führer Lajos	xx.10.1909		juvenile	Pelecaniformes
*Nycticoraxnycticorax*	Răscruci (CJ)	Führer Lajos	xx.10.1909		juvenile	Pelecaniformes
*Nycticoraxnycticorax*	Răscruci (CJ)	Führer Lajos	xx.10.1909		juvenile	Pelecaniformes
*Nycticoraxnycticorax*	Răscruci (CJ)	Führer Lajos	xx.10.1909		juvenile	Pelecaniformes
*Oenantheoenanthe*	Baciu (CJ)	Vincze Ferencz	25.07.1910	F	juvenile	Passeriformes
*Oenantheoenanthe*	Gilău (CJ)	Führer Lajos	xx.04.1911	M	adult	Passeriformes
*Oenantheoenanthe*	Gilău (CJ)	Führer Lajos	xx.04.1911	M	adult	Passeriformes
*Oenantheoenanthe*	Gilău (CJ)	Führer Lajos	xx.05.1911	M	adult	Passeriformes
*Oenantheoenanthe*	Gilău (CJ)	Führer Lajos	xx.05.1911	M	adult	Passeriformes
*Oenantheoenanthe*	Păniceni (CJ)	Führer Lajos	xx.04.1911	M	adult	Passeriformes
*Oenantheoenanthe*	Păniceni (CJ)	Führer Lajos	xx.04.1911	M	adult	Passeriformes
*Oenantheoenanthe*	Păniceni (CJ)	Führer Lajos	xx.04.1911	M	adult	Passeriformes
*Oenantheoenanthe*	Păniceni (CJ)	Führer Lajos	xx.04.1911	F	adult	Passeriformes
*Oenantheoenanthe*	Vița (CJ)	Führer Lajos	xx.09.1912	F	adult	Passeriformes
*Oriolusoriolus*	București (B)	Vincze Ferencz	12.05.1969	F	adult	Passeriformes
*Oriolusoriolus*	Brăncovenești (MS)	Vincze Ferencz	12.05.1971	M	adult	Passeriformes
*Oriolusoriolus*	Lita (CJ)	Vincze Ferencz	16.05.1983	M	adult	Passeriformes
*Otusscops*					adult	Strigiformes
*Otusscops*					adult	Strigiformes
*Otusscops*					adult	Strigiformes
*Parusmajor*	Cluj-Napoca (CJ)	Führer Lajos	30.10.1902	F		Passeriformes
*Parusmajor*	Cluj-Napoca (CJ)	Ajtai K. Gyula	20.01.1910	M		Passeriformes
*Parusmajor*	Cluj-Napoca (CJ)	Ajtai K. Gyula	20.01.1910	M		Passeriformes
*Parusmajor*	Cluj-Napoca (CJ)	Führer Lajos	xx.12.1909	M		Passeriformes
*Parusmajor*	Cluj-Napoca (CJ)	Führer Lajos	xx.11.1909	M		Passeriformes
*Parusmajor*	Cluj-Napoca (CJ)	Führer Lajos	xx.11.1909	M		Passeriformes
*Parusmajor*						Passeriformes
*Parusmajor*				F		Passeriformes
*Parusmajor*				F		Passeriformes
*Passerdomesticus*	Sucutard (CJ)	Fülöp Herman	31.01.1960	F	adult	Passeriformes
*Passerdomesticus*	Sucutard (CJ)	Fülöp Herman	31.01.1960	F	adult	Passeriformes
*Passerdomesticus*	Sucutard (CJ)	Fülöp Herman	31.01.1960	M	adult	Passeriformes
*Perdixperdix*	Baciu (CJ)	Vincze Ferencz	12.05.1970	F		Galliformes
*Periparusater*	Albac (AB)		xx.03.1913	M		Passeriformes
*Periparusater*	Albac (AB)		xx.03.1913	M		Passeriformes
*Periparusater*	Albac (AB)		xx.03.1913	F		Passeriformes
*Periparusater*	Cluj-Napoca (CJ)	Führer Lajos	xx.09.1909	M		Passeriformes
*Periparusater*	Cluj-Napoca (CJ)	Führer Lajos	xx.12.1909	F		Passeriformes
*Periparusater*	Cluj-Napoca (CJ)	Führer Lajos	xx.12.1909	F		Passeriformes
*Periparusater*	Cluj-Napoca (CJ)	Führer Lajos	xx.12.1909	M		Passeriformes
*Periparusater*	Cluj-Napoca (CJ)	Führer Lajos	xx.12.1909	M		Passeriformes
*Periparusater*	Cluj-Napoca (CJ)	Führer Lajos	xx.12.1909	M		Passeriformes
*Periparusater*	Cluj-Napoca (CJ)	Führer Lajos	xx.11.1909	M		Passeriformes
*Periparusater*						Passeriformes
*Periparusater*						Passeriformes
*Periparusater*						Passeriformes
*Periparusater*						Passeriformes
*Pernisapivorus*				M	adult	Accipitriformes
*Phoenicurusochruros*	Feleacu (CJ)	Führer Lajos	xx.04.1911	M	adult	Passeriformes
*Phoenicurusochruros*	Feleacu (CJ)	Führer Lajos	xx.04.1911	M	adult	Passeriformes
*Phoenicurusochruros*	Feleacu (CJ)	Führer Lajos	xx.04.1911	M	adult	Passeriformes
*Phoenicurusphoenicurus*	Cluj-Napoca (CJ)	Vincze Ferencz	26.05.1965	M	adult	Passeriformes
*Phoenicurusphoenicurus*	Cuzăplac (SJ)	Kómis Lajos	02.12.1903	F		Passeriformes
*Phoenicurusphoenicurus*	Feleacu (CJ)	Führer Lajos	xx.04.1911	M		Passeriformes
*Phoenicurusphoenicurus*	Feleacu (CJ)	Führer Lajos	xx.04.1911	M		Passeriformes
*Phoenicurusphoenicurus*	Gilău (CJ)	Führer Lajos	xx.10.1909	M	juvenile	Passeriformes
*Phoenicurusphoenicurus*				M		Passeriformes
*Phoenicurusphoenicurus*						Passeriformes
*Phoenicurusphoenicurus*						Passeriformes
*Phoenicurusphoenicurus*				M		Passeriformes
*Phylloscopuscollybita*						Passeriformes
*Phylloscopuscollybita*	Florești (CJ)	Führer Lajos	xx.04.1913	M	adult	Passeriformes
*Phylloscopuscollybita*	Cluj-Napoca (CJ)	Führer Lajos	xx.10.1909	M		Passeriformes
*Phylloscopuscollybita*	Cluj-Napoca (CJ)	Führer Lajos	xx.10.1909	M		Passeriformes
*Phylloscopuscollybita*	Cluj-Napoca (CJ)	Führer Lajos	xx.10.1909	F		Passeriformes
*Phylloscopuscollybita*	Cluj-Napoca (CJ)	Führer Lajos	xx.09.1912		juvenile	Passeriformes
*Phylloscopuscollybita*	Feleac (CJ)	Führer Lajos	xx.05.1911	F		Passeriformes
*Phylloscopuscollybita*	Feleac (CJ)	Führer Lajos	xx.05.1911	F		Passeriformes
*Phylloscopuscollybita*	Gilău (CJ)	Führer Lajos	xx.09.1909	M		Passeriformes
*Phylloscopuscollybita*	Gilău (CJ)	Führer Lajos	xx.09.1909	F		Passeriformes
*Phylloscopuscollybita*						Passeriformes
*Phylloscopuscollybita*						Passeriformes
*Phylloscopuscollybita*						Passeriformes
*Phylloscopuscollybita*						Passeriformes
*Phylloscopuscollybita*						Passeriformes
*Phylloscopuscollybita*						Passeriformes
*Phylloscopuscollybita*						Passeriformes
*Phylloscopuscollybita*						Passeriformes
*Picapica*	Cluj-Napoca (CJ)	Führer Lajos	xx.03.1912	M		Passeriformes
*Picapica*	Cluj-Napoca (CJ)	Zwörner Sándor	14.02.1904	M		Passeriformes
*Picapica*						Passeriformes
*Picuscanus*	Ciurila (CJ)	Führer Lajos	xx.11.1909	M	adult	Piciformes
*Picuscanus*	Cluj-Napoca (CJ)	Führer Lajos	xx.10.1912	F		Piciformes
*Picuscanus*	Cluj-Napoca (CJ)	Führer Lajos	xx.11.1909	F	adult	Piciformes
*Picuscanus*	Cuzăplac (SJ)	Kómis Lajos	03.12.1913	F	adult	Piciformes
*Picuscanus*				M	adult	Piciformes
*Picusviridis*				F	adult	Piciformes
*Picusviridis*				F	adult	Piciformes
*Platalealeucorodia*	Delta Dun?rii	Fülöp Herman	15.04.1950		adult	Pelecaniformes
*Platalealeucorodia*	Dobrogea	Führer Lajos	xx.xx.1911	M	adult	Pelecaniformes
*Platalealeucorodia*	Dobrogea	Führer Lajos	xx.xx.1911	F	adult	Pelecaniformes
*Platalealeucorodia*	Dobrogea	Führer Lajos	xx.xx.1911	M	adult	Pelecaniformes
*Platalealeucorodia*	Dobrogea	Führer Lajos	xx.xx.1911	M	adult	Pelecaniformes
*Platalealeucorodia*	Dobrogea	Führer Lajos	xx.xx.1911	F	adult	Pelecaniformes
*Platalealeucorodia*	Mociu (CJ)	Führer Lajos	xx.07.1910	M	adult	Pelecaniformes
*Podicepscristatus*	Cefa (BH)	Vincze Ferencz	26.06.1970	M		Podicipediformes
*Podicepscristatus*	Cefa (BH)	Vincze Ferencz	26.06.1970	M	adult	Podicipediformes
*Podicepscristatus*	Cefa (BH)	Vincze Ferencz	26.06.1970	M	adult	Podicipediformes
*Podicepscristatus*	Cefa (BH)	Vincze Ferencz	26.06.1970	M	adult	Podicipediformes
*Podicepscristatus*	Cluj-Napoca (CJ)	Zwörner Sándor	xx.10.1962	F	adult	Podicipediformes
*Podicepscristatus*						Podicipediformes
*Podicepsgrisegena*	Geaca (CJ)	Vincze Ferencz	03.06.1970	M	adult	Podicipediformes
*Podicepsnigricollis*	Apahida (CJ)	Führer Lajos	xx.10.1909	F		Podicipediformes
*Podicepsnigricollis*	Dej (CJ)	Varró Dezső	07.08.1944	M		Podicipediformes
*Poecilelugubris*	Cuzăplac (SJ)	Kómis Lajos	04.01.1914	M	adult	Passeriformes
*Poecilelugubris*	Gilău (CJ)	Führer Lajos	xx.06.1911	M		Passeriformes
*Poecilelugubris*	Gilău (CJ)	Führer Lajos	xx.06.1911	F	adult	Passeriformes
*Poecilelugubris*	Gilău (CJ)	Führer Lajos	xx.05.1911	F	adult	Passeriformes
*Poecilelugubris*					adult	Passeriformes
*Poecilepalustris*	Cluj-Napoca (CJ)	Führer Lajos	xx.12.1909	F		Passeriformes
*Poecilepalustris*	Cluj-Napoca (CJ)	Führer Lajos	xx.12.1909	M		Passeriformes
*Poecilepalustris*	Cluj-Napoca (CJ)	Führer Lajos	xx.12.1909	M		Passeriformes
*Poecilepalustris*	Cluj-Napoca (CJ)	Führer Lajos	15.10.1912	M		Passeriformes
*Poecilepalustris*	Cluj-Napoca (CJ)	Führer Lajos	15.10.1912	M		Passeriformes
*Poecilepalustris*	Cuzăplac (SJ)	Kómis Lajos	16.10.1913	F		Passeriformes
*Poecilepalustris*	Micești (CJ)	Führer Lajos	xx.11.1909	M		Passeriformes
*Poecilepalustris*	Micești (CJ)	Führer Lajos	xx.02.1910	F		Passeriformes
*Poecilepalustris*	Micești (CJ)	Führer Lajos	xx.02.1910	M		Passeriformes
*Poecilepalustris*						Passeriformes
*Poecilepalustris*						Passeriformes
*Poecilepalustris*						Passeriformes
*Poecilepalustris*						Passeriformes
*Pomatostomussuperciliosus*	Victoria (AU)	Gasilemaine	xx.09.1897			Passeriformes
*Porzanaporzana*	Apahida (CJ)	Führer Lajos	xx.10.1909	F	juvenile	Gruiformes
*Porzanaporzana*	Apahida (CJ)	Führer Lajos	xx.10.1909	M	juvenile	Gruiformes
*Porzanaporzana*	Apahida (CJ)	Zwörner Sándor	06.10.1903	M	juvenile	Gruiformes
*Porzanaporzana*	Florești (CJ)	Führer Lajos	xx.05.1911	M	adult	Gruiformes
*Porzanaporzana*	Mociu (CJ)	Führer Lajos	xx.11.1909	F	juvenile	Gruiformes
*Porzanaporzana*	Mociu (CJ)	Führer Lajos	xx.11.1909	M	juvenile	Gruiformes
*Porzanaporzana*	Mociu (CJ)	Führer Lajos	xx.11.1909	M	adult	Gruiformes
*Prunellacollaris*			xx.xx.1911			Passeriformes
*Prunellacollaris*			xx.xx.1911			Passeriformes
*Prunellacollaris*			xx.xx.1911			Passeriformes
*Prunellacollaris*			xx.xx.1911			Passeriformes
*Prunellacollaris*						Passeriformes
*Prunellamodularis*	Cuzăplac (SJ)	Kómis Lajos	16.10.1913	M	adult	Passeriformes
*Prunellamodularis*	Feleacu (CJ)	Führer Lajos	xx.05.1911	M	adult	Passeriformes
*Prunellamodularis*	Feleacu (CJ)	Führer Lajos	xx.05.1911	M	adult	Passeriformes
*Prunellamodularis*						Passeriformes
*Prunellamodularis*						Passeriformes
*Prunellamodularis*						Passeriformes
*Prunellamodularis*						Passeriformes
*Pycnonotuscafer*	Sindanglaya	Xántus János	30.06.1870			Passeriformes
*Pyrrhulaphyrrhula*	Cluj-Napoca (CJ)	Fülöp Herman	14.03.1954	F	adult	Passeriformes
*Pyrrhulaphyrrhula*	Cluj-Napoca (CJ)		14.03.1954	M	adult	Passeriformes
*Pyrrhulaphyrrhula*	Cluj-Napoca (CJ)		14.03.1955	M	adult	Passeriformes
*Pyrrhulaphyrrhula*	Cluj-Napoca (CJ)	Fülöp Herman	23.02.1960	M	adult	Passeriformes
*Pyrrhulaphyrrhula*				M	adult	Passeriformes
*Pyrrhulaphyrrhula*				M		Passeriformes
*Recurvirostraavosetta*	Someșeni (CJ)	Führer Lajos	xx.09.1912		juvenile	Charadriiformes
*Regulusignicapillus*	Cluj-Napoca (CJ)	Führer Lajos	xx.10.1909	F		Passeriformes
*Regulusignicapillus*	Cluj-Napoca (CJ)	Führer Lajos	xx.10.1909	F		Passeriformes
*Regulusignicapillus*	Feleacu (CJ)	Führer Lajos	xx.04.1911	M		Passeriformes
*Regulusignicapillus*	Feleacu (CJ)	Führer Lajos	xx.05.1911	M		Passeriformes
*Regulusregulus*	Cluj-Napoca (CJ)	Führer Lajos	xx.10.1909	M		Passeriformes
*Regulusregulus*	Cluj-Napoca (CJ)	Führer Lajos	xx.04.1911	M		Passeriformes
*Regulusregulus*	Cluj-Napoca (CJ)	Führer Lajos	xx.10.1909	M		Passeriformes
*Regulusregulus*	Cluj-Napoca (CJ)	Ajtai K. Gyula	20.01.1910	M		Passeriformes
*Regulusregulus*	Cluj-Napoca (CJ)	Führer Lajos	xx.12.1909	F		Passeriformes
*Regulusregulus*	Cluj-Napoca (CJ)	Führer Lajos	xx.09.1909	M		Passeriformes
*Regulusregulus*	Cluj-Napoca (CJ)	Führer Lajos	xx.09.1909	F		Passeriformes
*Regulusregulus*	Cluj-Napoca (CJ)	Führer Lajos	xx.09.1909	M		Passeriformes
*Regulusregulus*	Feleacu (CJ)	Führer Lajos	xx.04.1911	M		Passeriformes
*Regulusregulus*	Feleacu (CJ)	Führer Lajos	xx.04.1911	M		Passeriformes
*Regulusregulus*	Feleacu (CJ)	Führer Lajos	xx.05.1911	M		Passeriformes
*Regulusregulus*	Feleacu (CJ)	Führer Lajos	xx.05.1911	M		Passeriformes
*Regulusregulus*	Feleacu (CJ)	Führer Lajos	xx.05.1911	M		Passeriformes
*Regulusregulus*	Feleacu (CJ)	Führer Lajos	xx.04.1911	M		Passeriformes
*Regulusregulus*	Feleacu (CJ)	Führer Lajos	xx.05.1911	M		Passeriformes
*Regulusregulus*	Feleacu (CJ)	Führer Lajos	xx.04.1911	M		Passeriformes
*Regulusregulus*	Feleacu (CJ)	Führer Lajos	xx.04.1911	M		Passeriformes
*Regulusregulus*	Feleacu (CJ)	Führer Lajos	xx.xx.1911	M		Passeriformes
*Regulusregulus*	Gilău (CJ)	Führer Lajos	xx.09.1909	F		Passeriformes
*Regulusregulus*	Micești (CJ)	Führer Lajos	xx.11.1909	F		Passeriformes
*Regulusregulus*	Micești (CJ)	Führer Lajos	xx.11.1909	M		Passeriformes
*Regulusregulus*						Passeriformes
*Regulusregulus*						Passeriformes
*Regulusregulus*						Passeriformes
*Regulusregulus*						Passeriformes
*Regulusregulus*						Passeriformes
*Regulusregulus*						Passeriformes
*Regulusregulus*						Passeriformes
*Regulusregulus*						Passeriformes
*Remizpendulinus*	Ungaria (HU)	Führer Lajos	xx.06.1911	F		Passeriformes
*Remizpendulinus*	Ungaria (HU)	Führer Lajos	xx.06.1911	F		Passeriformes
*Remizpendulinus*	Ungaria (HU)	Führer Lajos	xx.06.1911	F		Passeriformes
*Remizpendulinus*	Ungaria (HU)	Führer Lajos	xx.06.1911	F		Passeriformes
*Remizpendulinus*	Ungaria (HU)	Führer Lajos	xx.06.1911	M		Passeriformes
*Remizpendulinus*	Ungaria (HU)	Führer Lajos	xx.06.1911	M		Passeriformes
*Remizpendulinus*	Ungaria (HU)	Führer Lajos	xx.06.1911	M		Passeriformes
*Remizpendulinus*	Ungaria (HU)	Führer Lajos	xx.06.1911	M		Passeriformes
*Saxicolarubetra*	Apahida (CJ)	Führer Lajos	xx.09.1909	M	adult	Passeriformes
*Saxicolarubetra*	Baciu (CJ)	Führer Lajos	26.05.1910	F	adult	Passeriformes
*Saxicolarubetra*	Florești (CJ)	Führer Lajos	xx.04.1913			Passeriformes
*Saxicolarubicola*	Apahida (CJ)	Führer Lajos	xx.09.1909	M	adult	Passeriformes
*Saxicolarubicola*	Cluj-Napoca (CJ)	Vincze Ferencz	18.03.1965	M	adult	Passeriformes
*Saxicolarubicola*	Cluj-Napoca (CJ)	Neuwirth János	14.03.1903	M	adult	Passeriformes
*Saxicolarubicola*	Cuzăplac (SJ)	Kómis Lajos	11.07.1913	F	juvenile	Passeriformes
*Saxicolarubicola*	Cuzăplac (SJ)	Kómis Lajos	11.07.1913	M	adult	Passeriformes
*Saxicolarubicola*	Fânațele Clujului (CJ)	Neuwirth János	xx.04.1903	F	adult	Passeriformes
*Scolopaxrusticola*	Feleacu (CJ)	Führer Lajos	xx.xx.1911			Charadriiformes
*Sittaeuropaea*	Cluj-Napoca (CJ)	Führer Lajos	xx.10.1912	M		Passeriformes
*Sittaeuropaea*	Cluj-Napoca (CJ)	Führer Lajos	xx.04.1911	M	adult	Passeriformes
*Sittaeuropaea*	Feleac (CJ)	Führer Lajos	xx.05.1911	M	adult	Passeriformes
*Sittaeuropaea*	Feleac (CJ)	Führer Lajos	xx.05.1911	M	adult	Passeriformes
*Spatulaquerquedula*	Geaca (CJ)	Fülöp Herman	29.03.1957	M	adult	Anseriformes
*Spatulaquerquedula*	Geaca (CJ)	Fülöp Herman	29.03.1957	f	adult	Anseriformes
*Spatulaquerquedula*	Apahida (CJ)	Führer Lajos	xx.04.1911			Anseriformes
*Spatulaquerquedula*	Someșeni (CJ)	Vincze Ferencz	26.07.1973	F		Anseriformes
*Spinusspinus*	Florești (CJ)	Ráthonyi Károly	16.12.1943	M		Passeriformes
*Sternahirundo*	Apahida (CJ)	Führer Lajos	xx.09.1909	F	adult	Charadriiformes
*Sternahirundo*	Apahida (CJ)	Führer Lajos	xx.09.1909	M	adult	Charadriiformes
*Sternulaalbifrons*					adult	Charadriiformes
*Streptopeliaturtur*	Cluj-Napoca (CJ)	Führer Lajos	xx.08.1912		adult	Columbiformes
*Streptopeliaturtur*	Gilău (CJ)	Führer Lajos	xx.09.1909	M	adult	Columbiformes
*Streptopeliaturtur*					adult	Columbiformes
*Streptopeliaturtur*					adult	Columbiformes
*Streptopeliaturtur*					adult	Columbiformes
*Streptopeliaturtur*					adult	Columbiformes
*Streptopeliaturtur*					adult	Columbiformes
*Strixuralensis*	Jibou (SJ)	Zwörner Sándor	20.04.1903	F		Strigiformes
*Strixuralensis*						Strigiformes
*Sturnusvulgaris*	Colibița (BN)	Vincze Ferencz	06.05.1971	M	adult	Passeriformes
*Sturnusvulgaris*						Passeriformes
*Sylviaatricapilla*	Cluj-Napoca (CJ)	Führer Lajos	xx.09.1912	M		Passeriformes
*Sylviaatricapilla*	Cluj-Napoca (CJ)	Führer Lajos	xx.09.1912	M		Passeriformes
*Sylviaatricapilla*	Cluj-Napoca (CJ)	Führer Lajos	xx.09.1912	M	juvenile	Passeriformes
*Sylviaatricapilla*	Făget (CJ)	Führer Lajos	xx.09.1909	F	juvenile	Passeriformes
*Sylviaatricapilla*	Feleacu (CJ)	Führer Lajos	xx.05.1911	M		Passeriformes
*Sylviaatricapilla*	Gilău (CJ)	Führer Lajos	xx.05.1911	M	adult	Passeriformes
*Sylviaatricapilla*	Gilău (CJ)	Führer Lajos	xx.05.1911	M	adult	Passeriformes
*Sylviaatricapilla*	Gilău (CJ)	Führer Lajos	xx.05.1911	M	adult	Passeriformes
*Sylviaatricapilla*	Gilău (CJ)	Führer Lajos	xx.10.1909	M	juvenile	Passeriformes
*Sylviaatricapilla*				M	adult	Passeriformes
*Sylviaatricapilla*				F		Passeriformes
*Sylviacommunis*						Passeriformes
*Sylviacommunis*	Baciu (CJ)	Ajtai K. Gyula	26.06.1910	M		Passeriformes
*Sylviacommunis*	Cluj-Napoca (CJ)	Führer Lajos	xx.06.1910	F		Passeriformes
*Sylviacommunis*	Florești (CJ)	Führer Lajos	xx.04.1913	M		Passeriformes
*Sylviacommunis*	Gilău (CJ)	Führer Lajos	xx.10.1909	M		Passeriformes
*Sylviacommunis*						Passeriformes
*Sylviacommunis*						Passeriformes
*Sylviacommunis*						Passeriformes
*Sylviacommunis*						Passeriformes
*Sylviacurruca*	Aghireș (CJ)	Führer Lajos	xx.10.1909	M		Passeriformes
*Sylviacurruca*	Cluj-Napoca (CJ)	Führer Lajos	xx.06.1911			Passeriformes
*Sylviacurruca*						Passeriformes
*Sylvianisoria*	Gilău (CJ)	Führer Lajos	xx.05.1911	M		Passeriformes
*Sylvianisoria*	Gilău (CJ)	Führer Lajos	xx.05.1911	F		Passeriformes
*Tachybaptusruficollis*	Geaca (CJ)	Vincze Ferencz	15.10.1970	M		Podicipediformes
*Tachybaptusruficollis*	Geaca (CJ)	Vincze Ferencz	30.08.1971	F		Podicipediformes
*Tachybaptusruficollis*	Hăghig (CV)		28.12.1902	F		Podicipediformes
*Tachybaptusruficollis*	Mociu (CJ)	Führer Lajos	xx.10.1909	F		Podicipediformes
*Tachybaptusruficollis*	Mociu (CJ)	Führer Lajos	xx.11.1909	F		Podicipediformes
*Tachybaptusruficollis*	Mociu (CJ)	Führer Lajos	xx.11.1909	M		Podicipediformes
*Tringaerythropus*	Mociu (CJ)	Führer Lajos	xx.10.1909	M	adult	Charadriiformes
*Tringaerythropus*	Mociu (CJ)	Führer Lajos	xx.10.1909	M	adult	Charadriiformes
*Tringaerythropus*	Mociu (CJ)	Führer Lajos	xx.10.1909	F	adult	Charadriiformes
*Tringaglareola*	Gilău (CJ)	Führer Lajos	xx.05.1911	M	adult	Charadriiformes
*Tringaglareola*	Someșeni (CJ)	Vincze Ferencz	24.07.1973	M	juvenile	Charadriiformes
*Tringaglareola*					adult	Charadriiformes
*Tringanebularia*	Mociu (CJ)	Führer Lajos	xx.11.1909	M		Charadriiformes
*Tringanebularia*	Mociu (CJ)	Führer Lajos	xx.10.1909	M		Charadriiformes
*Tringanebularia*	Mociu (CJ)	Führer Lajos	xx.10.1909	M		Charadriiformes
*Tringanebularia*	Mociu (CJ)	Führer Lajos	xx.10.1909	M		Charadriiformes
*Tringanebularia*	Mociu (CJ)	Führer Lajos	xx.10.1909	M		Charadriiformes
*Tringanebularia*	Mociu (CJ)	Führer Lajos	xx.11.1909	F		Charadriiformes
*Tringanebularia*						Charadriiformes
*Tringaochropus*	Cluj-Napoca (CJ)	Führer Lajos	xx.10.1909	M		Charadriiformes
*Tringaochropus*	Țaga (CJ)	Ajtai K. Gyula	04.05.1910	M		Charadriiformes
*Tringaochropus*						Charadriiformes
*Tringaochropus*						Charadriiformes
*Tringaochropus*					adult	Charadriiformes
*Tringaochropus*						Charadriiformes
*Tringatotanus*	Mociu (CJ)	Führer Lajos	xx.11.1909	F	juvenile	Charadriiformes
*Troglodytestroglodytes*	Cluj-Napoca (CJ)	Führer Lajos	xx.10.1909	M		Passeriformes
*Troglodytestroglodytes*	Cluj-Napoca (CJ)	Führer Lajos	xx.10.1909	M		Passeriformes
*Troglodytestroglodytes*	Cluj-Napoca (CJ)	Führer Lajos	xx.12.1912	M		Passeriformes
*Troglodytestroglodytes*	Cluj-Napoca (CJ)	Führer Lajos	10.10.1912	M		Passeriformes
*Troglodytestroglodytes*	Cluj-Napoca (CJ)	Führer Lajos	xx.12.1912	M		Passeriformes
*Troglodytestroglodytes*	Cluj-Napoca (CJ)	Führer Lajos	xx.11.1909	M		Passeriformes
*Troglodytestroglodytes*	Cuzăplac (SJ)	Kómis Lajos	03.11.1913	M	adult	Passeriformes
*Troglodytestroglodytes*	Cuzăplac (SJ)	Kómis Lajos	20.12.1913	F		Passeriformes
*Troglodytestroglodytes*	Florești (CJ)	Führer Lajos	xx.01.1913	M		Passeriformes
*Troglodytestroglodytes*						Passeriformes
*Troglodytestroglodytes*						Passeriformes
*Turdusiliacus*	Făget (CJ)	Führer Lajos	xx.10.1909	M		Passeriformes
*Turdusmerula*	Cuzăplac (SJ)	Kómis Lajos	14.10.1913	M	juvenile	Passeriformes
*Turdusmerula*	Viștea (CJ)	Ajtai K. Gyula	17.03.1910	M	adult	Passeriformes
*Turdusmerula*				F	adult	Passeriformes
*Turdusmerula*						Passeriformes
*Turdusmerula*	Dumitra (AB)	Osváth Gergely	28.03.2018	M		Passeriformes
*Turdusmigratorius*	California (USA)	Xántus János	xx.xx.1859	M		Passeriformes
*Turdusphilomelos*	Cluj-Napoca (CJ)	Führer Lajos	xx.10.1909	M		Passeriformes
*Turdusphilomelos*	Cluj-Napoca (CJ)	Zwörner Sándor	xx.10.1903	M		Passeriformes
*Turdusphilomelos*	Cluj-Napoca (CJ)	Führer Lajos	xx.10.1909	F		Passeriformes
*Turdusphilomelos*	Micești (CJ)	Führer Lajos	xx.11.1909	M		Passeriformes
*Turdusphilomelos*	Cluj-Napoca (CJ)	Vizauer Tibor Csaba	01.10.2021		juvenile	Passeriformes
*Turduspilaris*	Căpușul Mare (CJ)		24.03.1984	M		Passeriformes
*Turduspilaris*	Făget (CJ)		24.03.1954	F		Passeriformes
*Turduspilaris*	Făget (CJ)		24.03.1954			Passeriformes
*Turduspilaris*					adult	Passeriformes
*Turdustorquatus*	Ceahlău (NT)	Vincze Ferencz	09.05.1971	F		Passeriformes
*Turdusviscivorus*	Borșa (CJ)	Kómis Lajos	26.12.1913	M		Passeriformes
*Turdusviscivorus*	Cluj-Napoca (CJ)	Führer Lajos	xx.10.1912	M		Passeriformes
*Turdusviscivorus*	Cluj-Napoca (CJ)	Führer Lajos	xx.10.1912	F		Passeriformes
*Turdusviscivorus*	Cluj-Napoca (CJ)	Führer Lajos	xx.02.1913	F		Passeriformes
*Turdusviscivorus*	Cluj-Napoca (CJ)	Zwörner Sándor	08.01.1903	F		Passeriformes
*Turdusviscivorus*	Cluj-Napoca (CJ)	Führer Lajos	08.01.1913	M		Passeriformes
*Turdusviscivorus*	Cluj-Napoca (CJ)	Führer Lajos	10.01.1913	F		Passeriformes
*Turdusviscivorus*	Cluj-Napoca (CJ)	Führer Lajos	05.01.1913	M		Passeriformes
*Turdusviscivorus*	Cuzăplac (SJ)	Kómis Lajos	21.12.1913	F		Passeriformes
*Turdusviscivorus*						Passeriformes
*Upupaepops*	Aghireș (CJ)	Führer Lajos	xx.09.1909	M		Bucerotiformes
*Upupaepops*	Aghireș (CJ)	Führer Lajos	xx.09.1909	F		Bucerotiformes
*Upupaepops*	Cluj-Napoca (CJ)	Ajtai K. Gyula	26.05.1910	F		Bucerotiformes
*Upupaepops*	Gilău (CJ)	Führer Lajos	xx.09.1909	F		Bucerotiformes
*Upupaepops*						Bucerotiformes
*Vanellusvanellus*	Cătina (CJ)	Zwörner Sándor	04.10.1903	M	juvenile	Charadriiformes
*Vanellusvanellus*	Hortobágy (HU)	Nagy Jenő	xx.04.1907	M	adult	Charadriiformes
*Vanellusvanellus*	Hortobágy (HU)	Nagy Jenő	xx.04.1907	F	adult	Charadriiformes
*Zaporniaparva*	Mociu (CJ)	Führer Lajos	xx.11.1909	M	juvenile	Gruiformes
*Zaporniaparva*	Mociu (CJ)	Führer Lajos	xx.11.1909	F	juvenile	Gruiformes
*Zaporniaparva*	Mociu (CJ)	Führer Lajos	xx.11.1909	M	juvenile	Gruiformes
*Zaporniaparva*	Răscruci (CJ)	Führer Lajos	xx.10.1909	M	juvenile	Gruiformes
*Zaporniaparva*	Ungaria (HU)	Führer Lajos	xx.05.1911	F	adult	Gruiformes
*Zaporniaparva*	Ungaria (HU)	Führer Lajos	xx.05.1911	F	adult	Gruiformes

**Figure 3.**
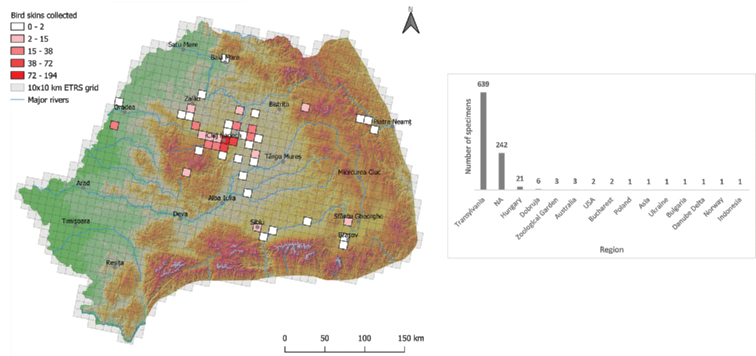
**a** The geographical distribution of the bird skin collection in the Zoological Museum of Babeș-Bolyai University with the number of bird specimens collected from each region (NA=data not available) **b** localities of birds collected in Transylvania.

The origins of 242 of 925 bird skin specimens are unknown, while three specimens were procured from different zoological gardens. 93.55% (639 out of 683) of specimens with known data were collected from Transylvania; only 43 specimens were collected outside this region, from different parts of Romania, Hungary, Ukraine, Bulgaria, Poland, and Norway (Fig. 3a, b). The collection includes eight exotic specimens: one with unknown data (*Melopsittacusundulatus*), three from Australia (*Eupsaltriaaustralis*, *Lichenostomusfuscus*, *Pomatostomussuperciliosus*), two from the USA (*Myiarchuscinerascens*, *Turdusmigratorius*) one from Indonesia (*Pycnonotuscafer*), and one collected from Transylvania that is often kept as pet (*Estrildatroglodytes*) (Table 1; Suppl. material 1: Table S1).

**Figure 4.**
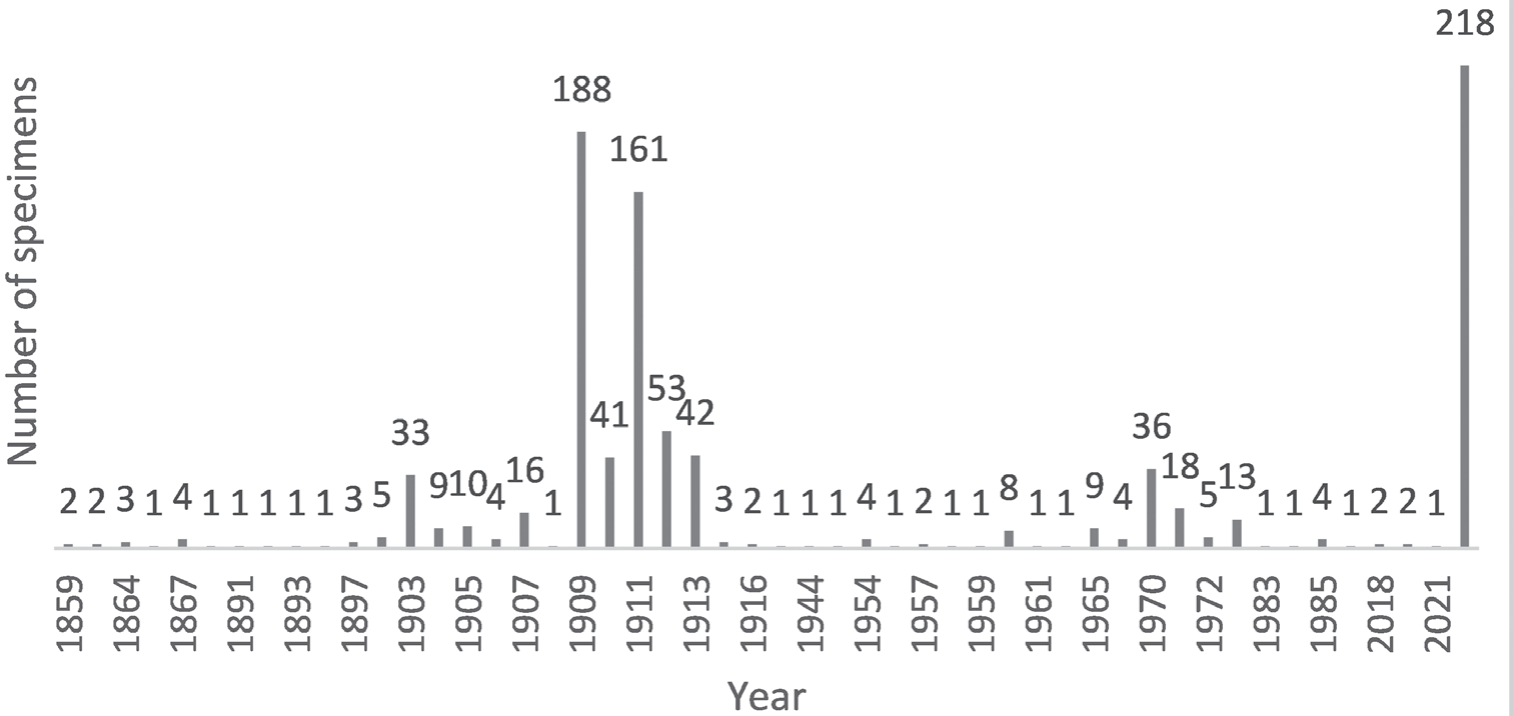
Temporal distribution of numbers of bird skin specimens added to the Zoological Museum of Babeș-Bolyai University Cluj-Napoca, Romania. (NA=218).

The bird skin specimens in the collection housed in the Zoological Museum of Babeș-Bolyai University were collected between 1859 and 2021 (Fig. 4). However, we were unable to identify the year of collection/acquisition for 218 individuals. The oldest specimens were collected by János Xántus during his Californian expeditions between 1857 -1859, and donated to the museum in 1959 (Frivaldszky 1865). The oldest native species in the collection with known data were collected by Ottó Herman in 1867. Most of the specimens were collected between 1909 and 1913 by Lajos Führer (460 specimens), followed by Ferencz Vincze (72 specimens) between 1970 and 1973. In total, the bird skin collection has had 26 different contributors (Table 1; Suppl. material 1: Table S1).

The collection also includes rarities and important avifaunistic data, for example one specimen of the Cinereous Vulture *Aegypiusmonachus*, two specimens of the Eastern Imperial Eagle *Aquilaheliaca*, and one Lesser Kestrel *Falconaumanni*, all collected between 1903 and 1907 from Transylvania (Table 1). The full catalogue of bird skin collection is provided in the Suppl. material 1: Table S1.

## Supplementary materials

Supplementary material 1 The catalogue of bird skin specimens held by the Zoological Museum of Babeş- Bolyai University, Cluj-Napoca, Romania Data type xslx. file. Explanation note The catalogue of bird skin specimens held by the Zoological Museum of Babeş-Bolyai University, Cluj-Napoca, Romania, including their new and ols inventory numbers, updated species identification and scientific name, locality, country and region of collection, gps coordinates, name of collector, date of collection, sex and age of birds. https://binary.pensoft.net/file/686211This dataset is made available under the Open Database License (http://opendatacommons.org/licenses/odbl/1.0/). The Open Database License (ODbL) is a license agreement intended to allow users to freely share, modify, and use this Dataset while maintaining this same freedom for others, provided that the original source and author(s) are credited.Gergely Osváth, Edgár Papp, Zoltán Benkő, Zsolt Kovács
